# A convenient approach to synthesize substituted 5-Arylidene-3-*m-*tolyl thiazolidine-2, 4-diones by using morpholine as a catalyst and its theoretical study

**DOI:** 10.1371/journal.pone.0247619

**Published:** 2021-03-04

**Authors:** Khorshada Jahan, Kaif Rashid Khan, Kawsari Akhter, Umme Kulsum Rowzatur Romman, Ershad Halim

**Affiliations:** Department of Chemistry, University of Dhaka, Dhaka, Bangladesh; Aligarh Muslim University, INDIA

## Abstract

Thiazolidinediones are very important and used as a drug for the treatment of type 2 diabetes. Here, we report a convenient approach to synthesis 3-m-tolyl-5-arylidene-2,4-thiazolidinediones (TZDs) derivatives **7a-e** in two steps with moderate to good yield using morpholine as a catalyst. All the structures were confirmed by their spectral IR, ^1^H NMR and ^13^C NMR data. The anti-diabatic activity of all synthesized molecules is evaluated by docking with peroxisome proliferator-activated receptor-γ (PPARγ). Preliminary flexible docking studies reveals that our compounds **7a**, **7d** and **7e** showed better binding affinity with the protein and could be a potential candidate for the treatment of type 2 diabetes in near future.

## Introduction

**Fig 1:**
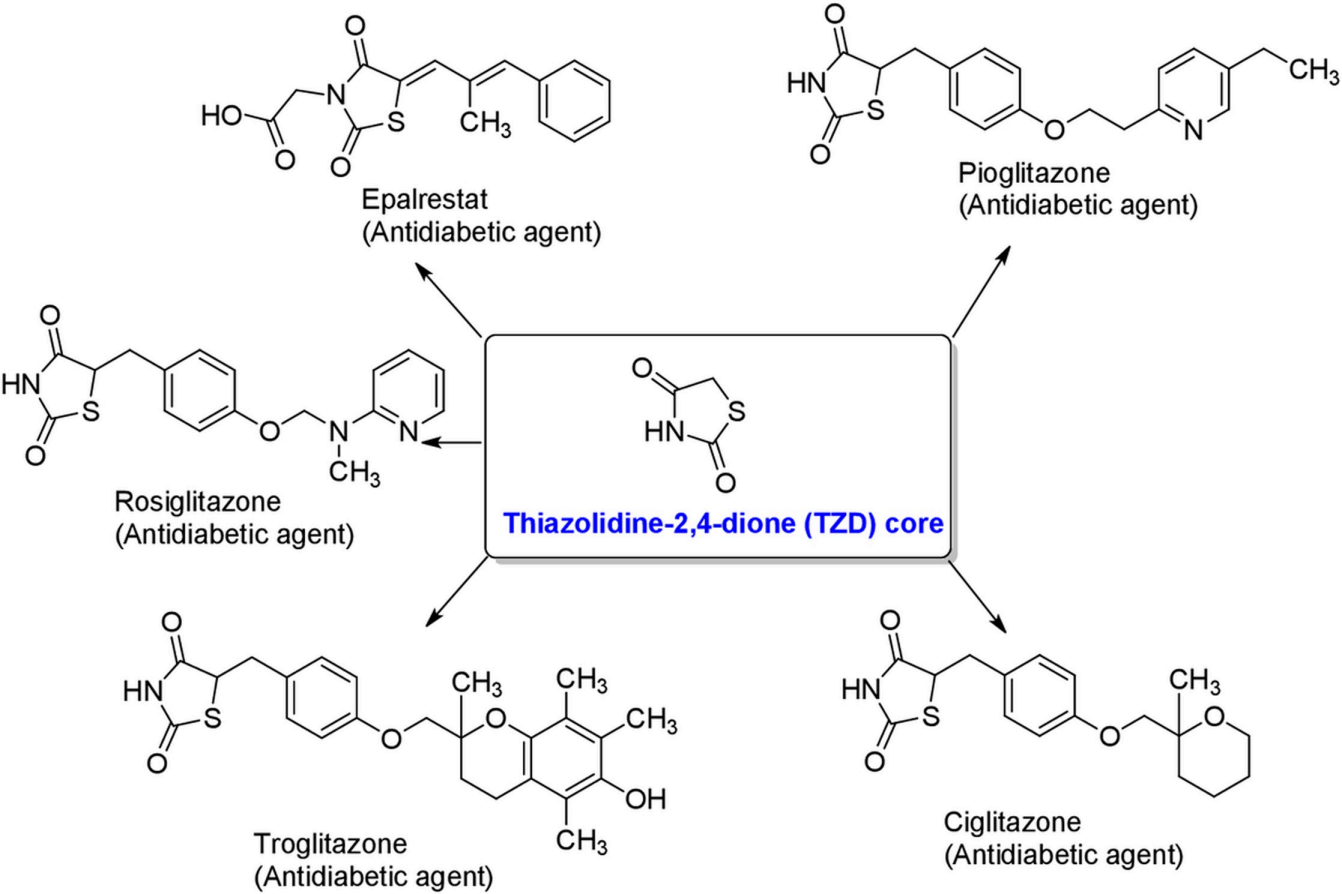
Thiazolidine-2,4-dione (TZD) and its derivatives.

Heterocyclic compounds play an important role in medicinal chemistry. Specifically, nitrogen-containing heterocycles with a sulfur atom such as, thiazolidine-2,4-dione (TZDs, **Fig 1**). TZDs represent an important class of compounds showed a wide spectrum of biological and pharmacological activities [1,2]. In the past decades, TZDs have been the subject of extensive research because of their involvement in the regulation of different physiological processes which has been confirmed by numerous reviews [3,4]. A variety of compounds having TZDs core (**Fig 1**) were used for the treatment of type II diabetes and related diseases [5]. Thiazolidinediones showed antidiabetic activity by binding with gamma form of the peroxisome proliferator-activated receptor-γ (PPARγ). This stimulates peripheral adiposities to increase their uptake of free fatty acids, which leads to reduction in the fat stored in muscles, liver and visceral fat deposits. The TZDs also leads to an increase in the secretion of adiponectin and a decrease in the production of inflammatory mediators such as tumor necrosis factor-alpha (TNF-α), plasminogen activator inhibitor-1(PAI-1) and interleukin-6 (IL-6). This feature lead TZDs act as an aldose reductase inhibitor and tumor inhibitor [6,7]. Moreover, chemical modification on this heterocycle led to a class of compounds that possess several biological activities including, anti-inflammatory effects [8], antibacterial [9–12], antitubercular [13], and antifungal activity [9]. TZDs target vascular cells and monocytes/macrophages to inhibit the production of pro-inflammatory cytokines [14–17] as well as the development of oxidative stress [18] and cell adhesion molecules. They are also key intermediates for the synthesis of anti-HIV and anti-ischemic agents [19–21]. Therefore, simple structure and valuable pharmacological activity of these molecules gained special attention from synthetic chemists and pharmacologists [20].

Several methods have been reported up to date for the synthesis of thiazolidine-2,4-dione and its derivatives with an extensive range of catalysts including Alum [22], glycine, sodium carbonate, piperidinium acetate [23], piperidine [24], amines salts [25], baker’s yeast [26], ionic liquids [27–31] and nanoparticle-supported copper (II) catalysts [32]. However, several of these existing methods suffer from one or more drawbacks, such as low yields, long reaction times, environmentally unfavorable solvents, and a requirement for excess catalyst. Hence, a facile efficient process is still desirable. As a continuation our research goal to develop a convenient method to synthesize valuable synthon [33–36] from easily available starting materials, we developed a two-step method to synthesize thiazolidine-2,4-dione derivatives by using morpholine as a catalyst. We have performed Density functional theory (DFT) calculations in all our synthesized molecules to find out most active compound. To the best of our knowledge, the use of morpholine as a catalyst for the synthesis of 3-*m*-tolyl-5-arylidenthiazolidine- 2,4-diones as well as DFT calculations has not been previously reported.

## Experimental

### Chemistry

#### General

All products were characterized by IR, UV, ^1^H-NMR and ^13^C-NMR. Thin layer chromatography (TLC) was carried out on plates coated with silica gel (Merck, Silica Gel G) and spots were detected with iodine vapour. The UV spectra was recorded using SHIMADZU UV-160A spectrophotometer using chloroform as solvent. Infra-Red spectra was recorded using SHIMADZU IR-470A spectrophotometer by direct transmittance using KBr pellets. Multinuclear NMR (^1^H, ^13^C, DEPT-135) spectra were recorded on a BRUKER 400 MHz NMR spectrophotometer. Chemical shifts are reported in parts per million (ppm). Tetramethyl silane (TMS) served as an internal standard in ^1^H and ^13^C NMR (δ 0.00 ppm). All the chemicals used for the synthesis of target compounds have been purchased from Sigma Aldrich and were used as received. Scanned Spectra for all compounds are shown in **S1–S29 Figs.**

#### General procedure for the synthesis of 3-(*m*-tolyl)thiazolidine-2,4-dione (4)

A solution of *m*-tolylthiourea, (**1**, 5.0g, 30.0 mmol), 2-chloroacetic acid, (**2**, 2.8 g, 30.0 mmol) and 20mL hydrochloric acid **3** (30%, (ν/ν)) were stirred for half an hour and then refluxed for overnight at 120°C in a 250mL round-bottomed flask. After completion of the reaction, as monitored by TLC (2:1, Pet ether: Ethyl acetate), the reaction mixture was gradually cooled to room temperature. Then it was neutralized with dilute sodium hydroxide (0.1 M). Immediately a solid mass was formed which was filtered under reduce pressure, washed with cold water (50mL) and recrystallized from absolute alcohol to give a pure product **4** as a white crystal (2.49g, 40% yield).

#### General procedure for the synthesis of 5-arylidene-3-(*m*-tolyl)-thiazolidine-2,4-diones (7a-e)

For each experiment, 20mmol of compound **4** and 20 mmol of substituted benzaldehydes **5a-e** was dissolved in absolute alcohol (10 mL) and refluxed with the Dean-Stark attachment for 2 hours by using morpholine **6** (10 mol%) as a catalyst. After completion of the reaction, as judged by TLC, the reaction was quenched with crushed ice. The crude product was filtered under reduced pressure, washed with cold water and purified by recrystallization using absolute alcohol. The yield of the products **7a-e** ranged from 55–90%.

#### Spectral data

*3-(m-tolyl) thiazolidine-2*, *4- dione*, *Compound (4)*. Yield: 40%; R_f_: 0.81; white crystal; UV (λ_max_ nm): 274.50; IR (KBr) (ν_max_ cm^-1^): 1760, 1688 (C = O, TZD ring), 1521, 1198 (-CH_2_ stretch), 761; ^1^H NMR (CDCl_3_, 400MHz, δ ppm): 7.43–7.32 (m, 3H), 7.13 (d, *J* = 7.6 Hz, 1H), 4.17 (d, *J* = 1.6 Hz, 2H), 2.203, (s, 3H); ^13^C NMR (CDCl_3_, 100MHz, δ ppm): 170.56, 170.43, 136.00, 131.97, 131.36, 130.09, 128.28, 127.25, 33.99, 17.52.

*5-(2-Methoxybenzylidene)-3-m-tolyl thiazolidine-2*, *4- dione (7a)*. Yield: 84% (morpholine 11%); R_f_: 0.34; yellow crystals; UV (λ_max_ nm): 358.50; IR (KBr) (ν_max_ cm^-1^): 1742, 1690 (C = O, TZD ring); ^1^H NMR (CDCl_3_, 400 MHz, δ ppm): 8.37 (s, 1H), 7.54–6.99 (m, 7H), 3.94 (s, 3H), 2.24 (s, 3H); ^13^C NMR (CDCl_3_, 100 MHz, δ ppm): 167.43, 165.61, 158.56, 136.26, 132.39, 132.13, 131.29, 130.47, 130.33, 129.94, 129.53, 128.43, 127.14, 121.31, 120.94, 111.23, 55.54, 17.65.

*5-(2-Chlorobenzylidene)-3-m-tolyl thiazolidine-2*, *4- dione (7b)*. Yield: 88%; R_f_: 0.50; green crystals; UV (λ_max_ nm): 358.50; IR (KBr) (ν_max_ cm^-1^): 1734, 1675 (C = O, TZD ring); ^1^H NMR (CDCl_3_, 400 MHz, δ ppm): 8.35 (s, 1H), 7.65–7.21 (m, 7H), 2.26 (s, 3H); ^13^C NMR (CDCl_3_, 100 MHz, δ ppm): 166.59, 164.92, 136.20, 136.06, 131.85, 131.80, 131.47, 131.37, 130.69, 130.54, 130.11, 128.35, 127.34, 127.22, 124.42, 17.66.

*5-(2-Nitrobenzylidene)-3-m-tolyl thiazolidine-2*, *4- dione (7c)*. Yield: 67%; R_f_: 0.22; light brown crystals; UV (λ_max_ nm): 332.00 and 243.50; IR (KBr) (ν_max_ cm^-1^): 1690, 1607 (C = O TZD ring), 1352(-NO_2_); ^1^H NMR (CDCl_3_, 400 MHz, δ ppm): 7.99 (s, 1H), 7.50–7.20 (m, 7H), 2.45 (s, 3H); ^13^C NMR (CDCl_3_, 100 MHz, δ ppm): 166.97, 165.63, 141.47, 136.23, 134.60, 132.03, 131.33, 130.53, 130.39, 130.06, 130.01, 127.19, 120.07, 17.64.

*5-(3-Hydroxybenzylidene)-3-m-tolyl thiazolidine-2*, *4- dione*, *Compound (7d)*. Yield: 54%; R_f_: 0.45; dark-brown powder; UV (λ_max_ nm): 328.00;; ^1^H NMR (CDCl_3_, 400 MHz, δ ppm): 7.96 (s, 1H), 7.53 (s, 4H), 7.51–7.35 (m, 3H), 7.22–7.19 (d, *J* = 7.6 Hz, 1H), 2.24 (s, 3H); ^13^C NMR (CDCl_3_, 100 MHz, δ ppm): 166.36, 165.32, 136.83, 136.18, 132.95, 131.87, 131.73, 131.39, 130.13, 129.64, 128.36, 127.25, 121.97, 17.63.

*5-(4-Chlorobenzylidene)-3-m-tolyl thiazolidine-2*, *4- dione*, *Compound (7e)*. Yield: 74%; R_f_: 0.12; off-white crystals; UV (λ_max_ nm): 334.50; IR (KBr) (ν_max_ cm^-1^): 1857 and 1783 (C = O TZD ring); ^1^H NMR (CDCl_3_, 400 MHz, δ ppm): 7.96 (s, 1H), 7.52 (s, 4H), 7.43–7.37 (m, 3H), 7.21–7.19 (d, *J* = 7.6 Hz, 1H), 2.24 (s, 3H); ^13^C NMR (CDCl_3_, 100 MHz, δ ppm): 166.37, 165.32, 136.85, 136.17, 132.96, 131.84, 131.72, 131.38,131.36, 130.12, 129.64, 128.34, 127.24, 121.96, 17.61.

### Computational methods

#### DFT studies

All theoretical calculations were performed by using density functional theory (DFT), B3LYP (6-31G, d) basis set in Gaussian 09 Program suite. A full geometry optimization was performed for all structures, using this function and all geometries were visualized using Avogadro 1.2 software package.

#### Preparation of the protein

**Fig 2:**
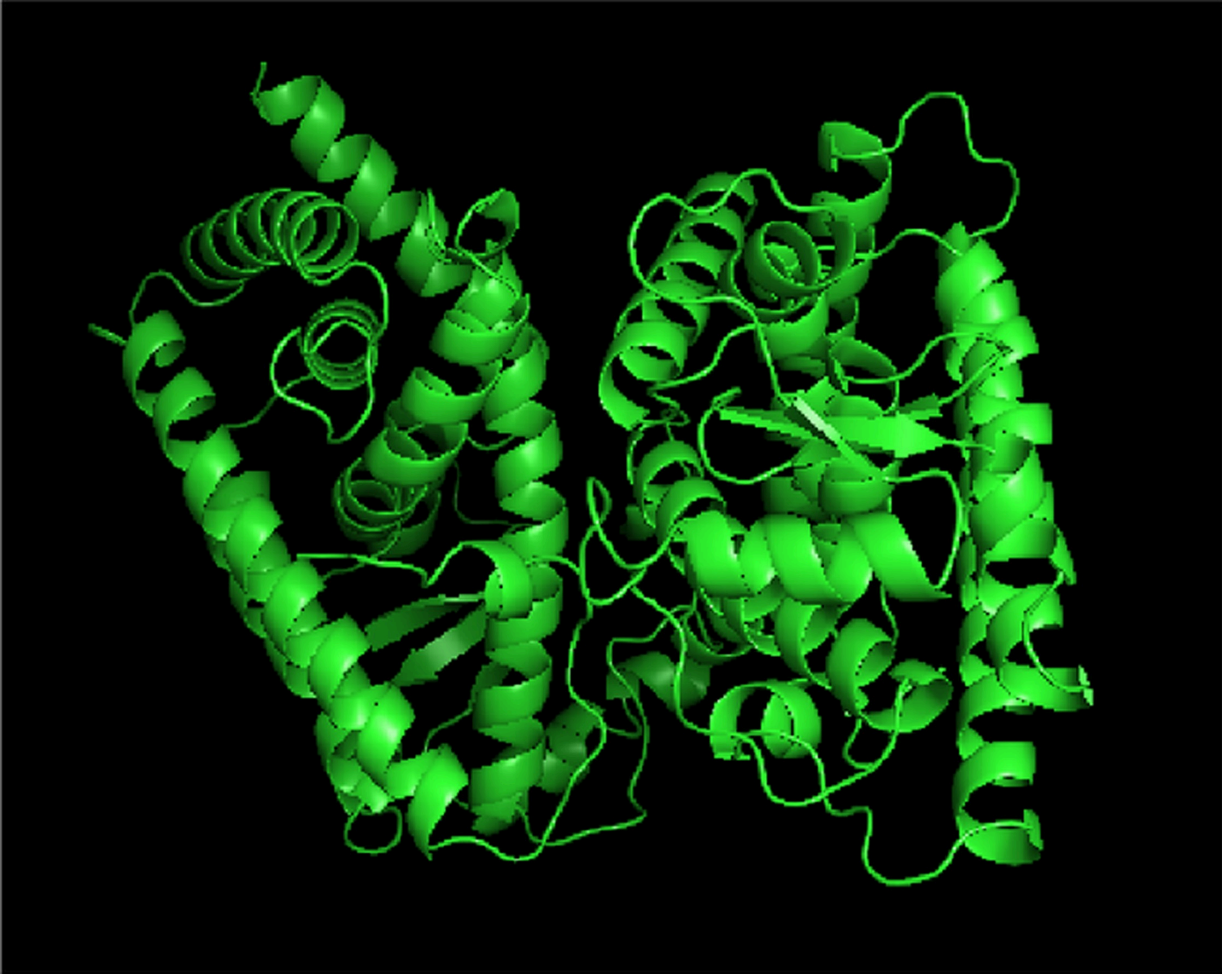
The X-ray crystal structure of PPARγ (PDB ID: 3GBK).

Crystal structure of human peroxisome proliferator-activated receptor gamma (PPARγ) [37] (PDB ID: 3GBK) was downloaded from RCSBPDB webpage (www.rcsb.org) and opened on PyMol (version 1.3) and waters and the agonist were removed from the crystal structure. The protein structure was then subjected to geometry and energy minimization in Swiss-PDBViewer (version 4.1.0) using GROMOS96 force field. The crystal structure of the protein after energy minimization was shown in Fig 2. The crystal structure was then saved as a.pdb file and used for molecular docking against the optimized structures of **4** and **7a-e**.

#### Molecular docking

Docking of the optimized structures were performed against 3GBK using AutoDock Vina. As a control, the commercially available type 2 anti-diabatic drug, Epalrestat was used. Only flexible docking was performed where the ligands were flexible, but the protein was held rigid. The dimension of the grid-box for the protein was set to 73.5450, 60.6299, 79.5208 Å as the X-, Y- and Z-coordinates respectively. After docking, all generated files were collected and their non-covalent interactions with the energy minimized protein was evaluated in Accelerys Discovery Studio 4.

## Results and discussion

### Chemistry

The synthesis of the new TZD derivatives had two steps. At first phenyl thiourea **1** reacts with chloroacetic acid **2** in the presence of hydrochloric acid **3** (30 mol%, (ν/ν)) as a catalyst to produce the desired 3-(*m*-tolyl) thiazolidine-2,4-dione **4**. Then the active methylene in position 5 of compound **4** undergo Knoevenagel condensations with various aromatic aldehydes **5a-e** in the presence of morpholine **6** to form compounds **7a–e** (S33 Fig). The yield in the first step is low because of the presence of unreacted starting material and formation of potential by product 3-toluidine (mechanism presented in **S30 Fig**). In second step, it should be noted that both electron-withdrawing and electron-donating groups in aromatic aldehydes are quite suitable and give the desired product good to moderate yield, except compound **7d**, where the presence of hydroxy group, the reaction become sluggish and it is difficult to separate the product.

After completing the reaction, all the products are recrystallized by using suitable solvent and checked by TLC. All the structures were confirmed by their spectral data (IR, UV, ^1^H NMR, and ^13^C NMR). In compound **4**, the IR spectra exhibited characteristic absorption bands at 1680–1690 cm^-1^ and 1755–1765 cm^-1^ due to the two C = O from the TZD heterocycle. The ^1^H NMR spectra showed characteristic doublets due to presence of C (5)-H in compound **4** at 4.169 and 4.173 ppm respectively which is further confirmed by DEPT spectra shown is **S5 Fig**. The condensation reaction between **4** and **5** formed compound **7**, where The C (5)–H in thiazole disappeared and a characteristic peak appeared around 8.0 ppm indicate the conversion of the methylene group to C = C double bond. Both ^13^C NMR and DEPT-135 data supported the conversion of the methylene group to C = C double bond.

### DFT studies: Thermodynamic results

Compounds **4** and **7a-e** were optimized to obtain their most thermodynamically favorable configuration where Epalrestat was used as a control compound. The thermodynamic data of all synthesized compounds have negative electronic energy, enthalpy and Gibbs-free energy suggesting that all the synthesized molecules are thermodynamically stable (**Table 1**). Compound **4** is thermodynamically less stable than compared to the other compounds **7a-e** including Epalrestat. In compounds **7a-e,** all compounds have similar energy except **7b** and **7e** which have lower energy because of the presence of Cl atom, hence, more stable. It should be noted that compounds **7a-e** after Knoevenagel condensation develop an alkene bond which stabilized the molecule by delocalized pi-orbitals hence favor lower energy. In order to find out the binding energy and hydrogen bonding capability, the dipole moment of all synthesized compounds was determined (**S31 Fig** and **Table 1**). The range of dipole moment for the synthesized molecules is in between 0.5–5.5 Debye, where **7e** has the lowest dipole moment because of the presence chlorine atom at *para*-position and most for Epalrestat because it contains polar groups such as a carboxyl.

**Table 1:** Theoretical thermodynamic results of Epalrestat, compound 4 and 7a-e.

Compound	Internal Energy, E	Enthalpy, ΔH	Free energy, ΔG	Dipole Moment, μ (D)
**Epalrestat**	-1333.010	-1333.009	-1333.080	5.5006
**4**	-989.721	-989.720	-989.774	1.0328
**7a**	-1373.259	-1373.258	-1373.332	4.1495
**7b**	-1718.372	-1718.371	-1718.443	3.0389
**7c**	-1463.259	-1463.258	-1463.333	5.1613
**7d**	-1333.984	-1333.983	-1334.053	1.3643
**7e**	-1718.376	-1718.375	-1718.446	0.4355

#### Electrostatic results

To find out the reactivity of the molecule towards its receptor, HOMO-LUMO and hardness and softness of all synthesized molecules were calculated (**S32 Fig and Table 2).** In the Frontier molecular orbital, large energy gap between HOMO-LUMO indicates that the molecule is more stable and less reactive, where low energy gap suggests easier electronic transition and favor quicker reaction. All the synthesized compounds **7a-e** except compound **4** have similar orbital energy gap suggesting similar reactivity towards its receptor.

**Table 2:** Theoretical electrostatic results of Epalrestat, compound 4 and 7a-e.

Compounds	HOMO (a.u)	LUMO (a.u)	Orbital energy gap (a.u)	Softness	Hardness
**Epalrestat**	-0.21876	-0.08698	0.13178	15.177	0.06589
**4**	-0.24558	-0.03353	0.21205	9.4312	0.10603
**7a**	-0.21447	-0.07913	0.13534	14.778	0.06767
**7b**	-0.23280	-0.08768	0.14512	13.782	0.07256
**7c**	-0.24179	-0.10205	0.13929	14.358	0.06965
**7d**	-0.22289	-0.08649	0.13640	14.663	0.06820
**7e**	-0.22956	-0.09098	0.13858	14.432	0.06929

### Docking studies

**Fig 3:**
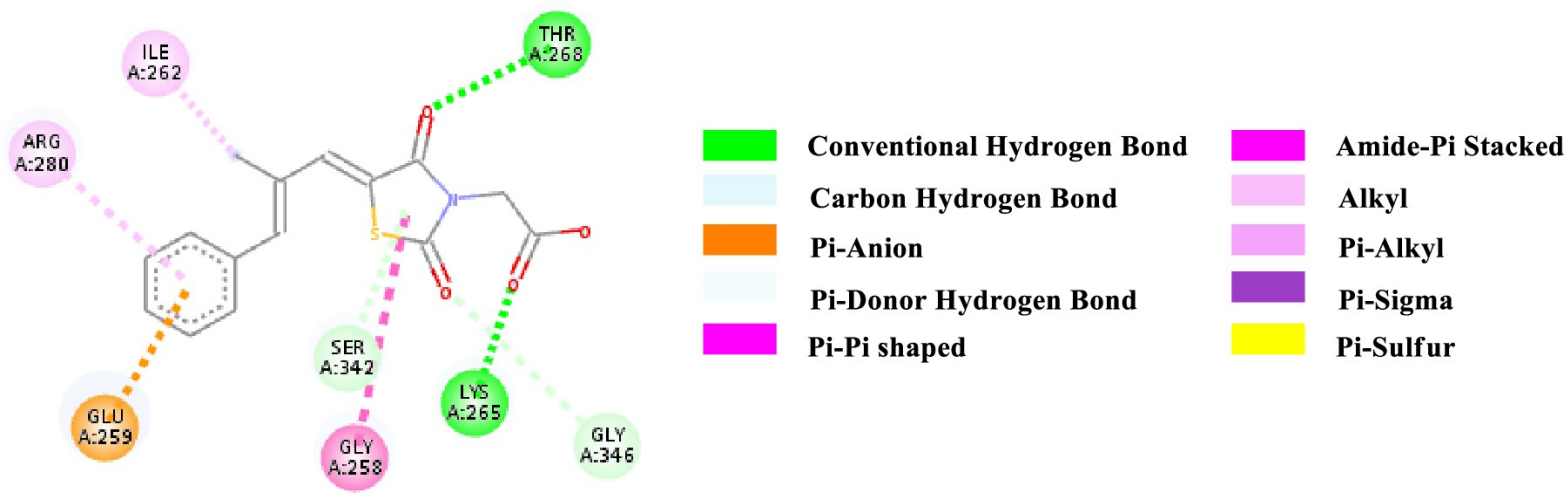
2D-representation of the non-covalent interactions of Epalrestat with receptor (3gbk) after flexible docking.

**Fig 4:**
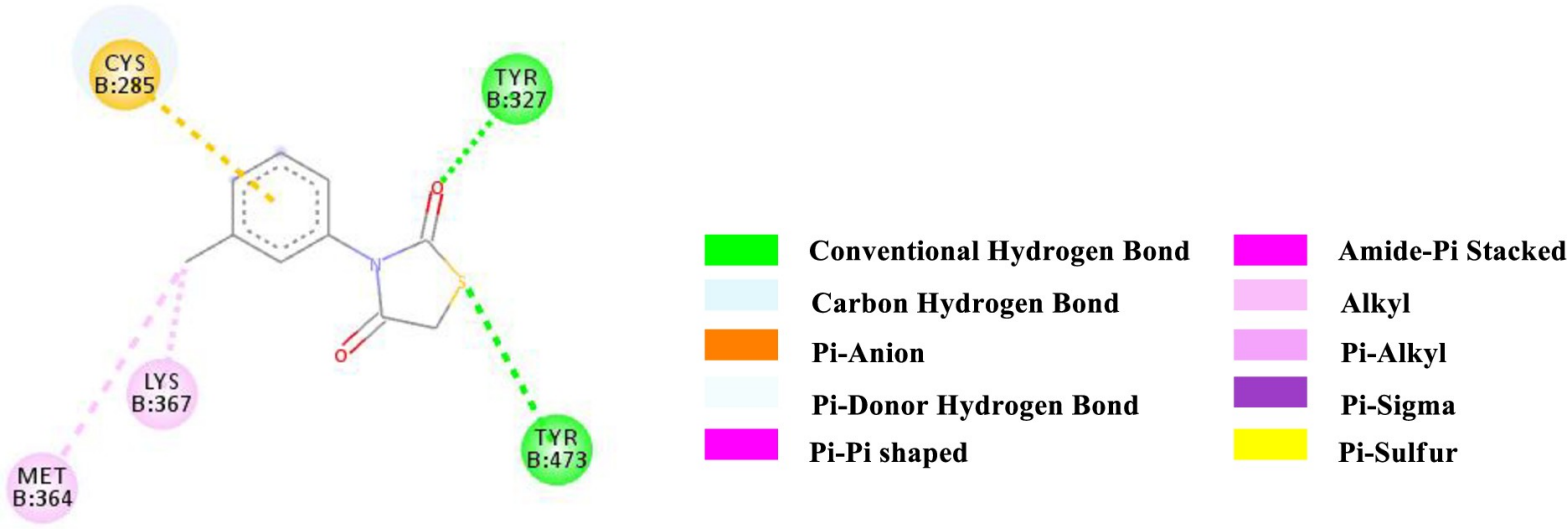
2D-representation of the non-covalent interactions of 4 and receptor (3gbk) after flexible docking.

**Fig 5:**
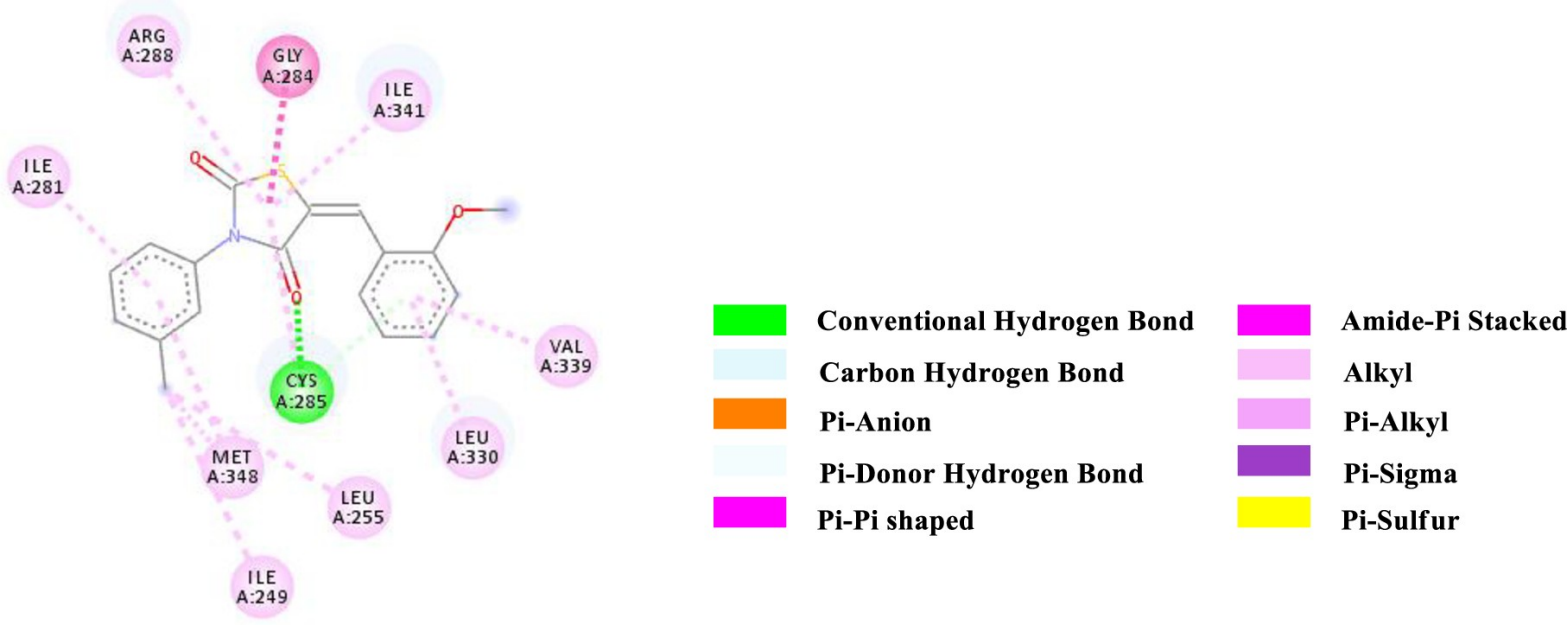
2D-representation of the non-covalent interactions of 7a with receptor (3gbk) after flexible docking.

**Fig 6:**
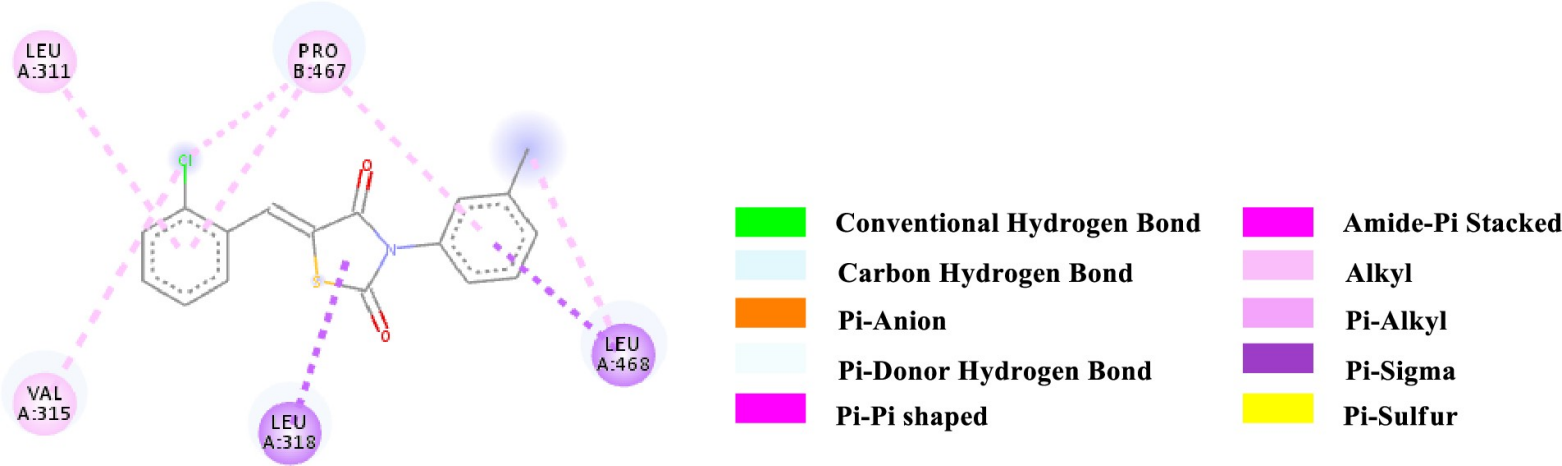
2D-representation of the non-covalent interactions of 7b with receptor (3gbk) after flexible docking.

**Fig 7:**
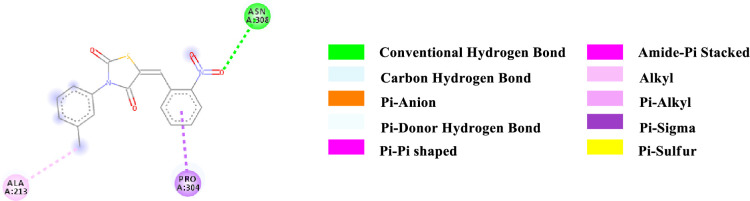
2D-representation of the non-covalent interactions of 7c with receptor (3gbk) after flexible docking.

**Fig 8:**
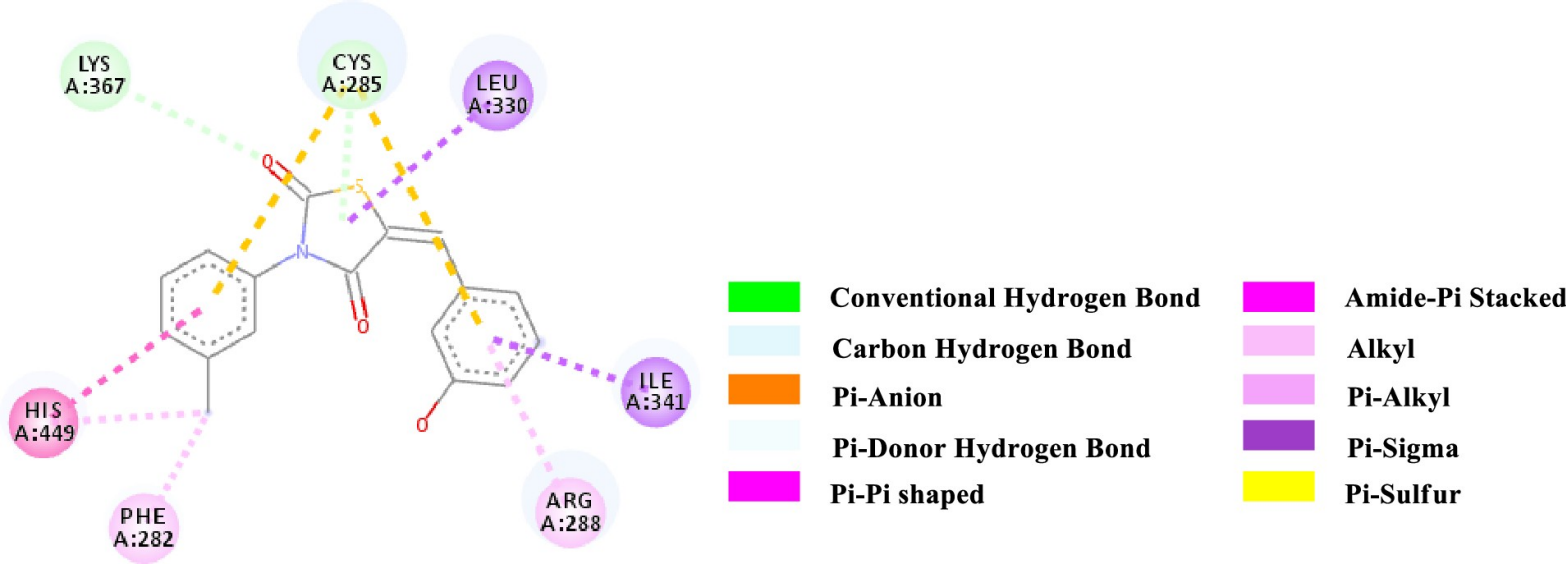
2D-representation of the non-covalent interactions of 7d with receptor (3gbk) after flexible docking.

**Fig 9:**
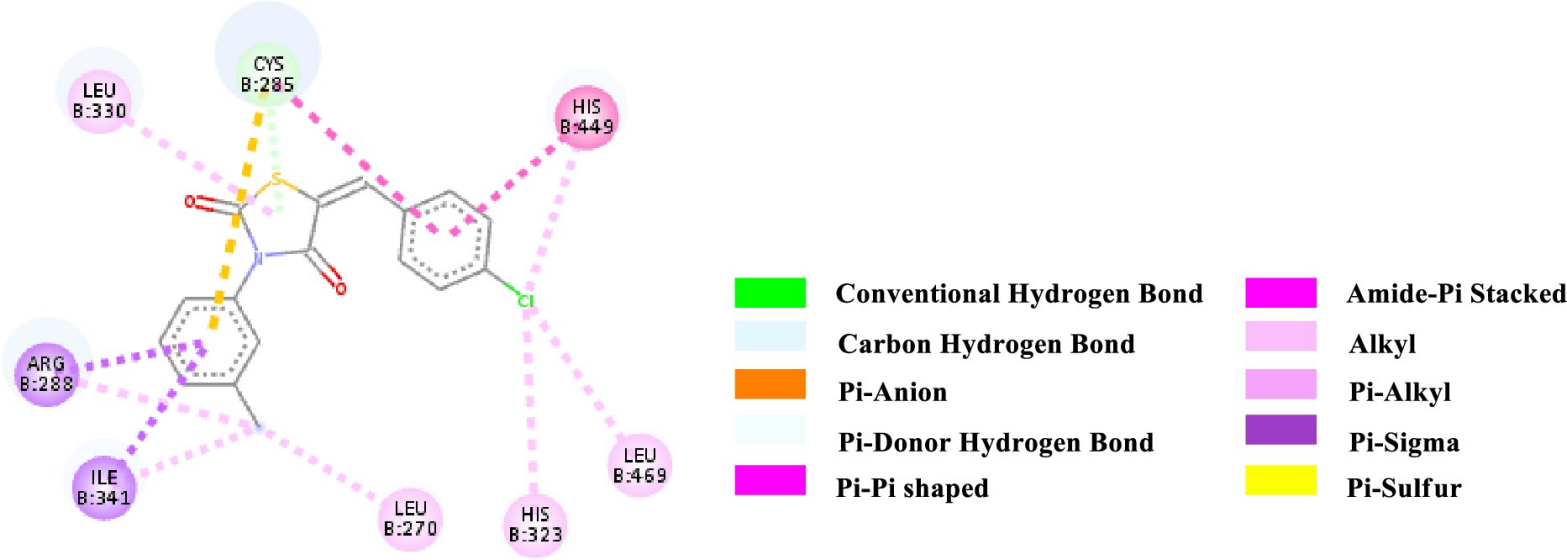
2D-representation of the non-covalent interactions of 7e with receptor (3gbk) after flexible docking.

Binding affinity is a measure of how strongly a molecule can fit into a receptor and interact with it through non-covalent bonding. To find out the binding affinity as well as protein interaction, we performed docking of all our synthesized compounds **4** and **7a-e** with Peroxisome proliferator-activated receptor gamma **(PPAR-γ or PPARG)** by considering Epalrestat as a reference (**Figs 3–9** and **Table 3**). In result, three compounds **7a**, **7d** and **7e** showed better binding affinity with the protein having energy -8.2, -8.5 and -8.9 kcal/mol respectively than compared to Epalrestat which has a binding affinity of -7.9 kcal/mol and hence showed better non-covalent interactions. Compound **4** has -6.9 kcal/mol of energy when bound to the receptor. This result indicates that the binding pocket of the protein is quite large so the structure bigger than **4** would have more interactions when bound to the protein. This was true for all our compounds **7a-e** which had higher binding affinity than **4**. However, Compound **7b** and **7c** have lower binding affinity than Epalrestat which indicate that the ortho position may not be a good site for better binding affinity. Only exception was observed with **7a,** where the methoxy group at ortho-position had no interaction with the protein. This result suggests that a good choice in a substituent may induce better binding. Compound **7e** has better binding where *p*-Cl atom interacts with the protein. This finding suggests that adding larger groups in the *para* position may lead to higher binding affinity.

**Table 3:** Binding affinities and types of non-bonding interactions of the optimized ligands Epalrestat, compound 4 and 7a-e with the protein.

Compounds	Binding Affinity, kcal/mol	Type of Non-bonding Interaction	From	To	Bond Distance, Å
**Epatrestat**	-7.9	HB	Lys 265 (NH)	C = O (ligand)	3.06067
		HB	Thr 268 (OH)	C = O (4^th^ position of TZD)	3.21220
		CHB	Gly 346 (αCH)	C = O (2^nd^ position of TZD)	3.64643
		PA	Glu 259 (COO^-^)	Benzene (ligand)	3.64293
		PDHB	Ser 342 (OH)	TZD core	3.72507
		APS	Gly 258—Glu 259 (CONH)	TZD core	4.20658
		Alkyl	Methyl (ligand)	Ile 262 (side chain)	5.38888
		Pi-Alkyl	Benzene (ligand)	Arg 280 (side chain)	4.88631
**4**	-6.1	HB	Tyr 327 (OH)	C = O (2^nd^ position of TZD)	3.12856
		HB	Tyr 473 (OH)	TZD (Sulfur)	3.31594
		PS	Cys 285 (SH)	N-Aryl (ligand)	4.25927
		Alkyl	Methyl (N-Aryl, ligand)	Met 364 (side chain)	5.29768
		Alkyl	Methyl (N-Aryl, ligand)	Lys 367 (side chain)	4.27732
**7a**	-8.2	HB	Cys 285 (SH)	C = O (4^th^ position of TZD)	3.71078
		PDHB; PS	Cys 285 (SH)	2-OMe (Aryl); TZD core (ligand)	4.11671
		APS	Gly 284; Cys 285 (CONH)	TZD core (ligand)	3.90759
		Alkyl	Methyl (Aryl; ligand)	Ile 249 (side chain)	4.68460
		Alkyl	Methyl (Aryl; ligand)	Leu 255 (side chain)	5.06828
		Alkyl	Methyl (Aryl; ligand)	Met 348 (side chain)	5.05667
		Pi-Alkyl	2-OMe (Aryl; ligand)	Cys 285 (side chain)	5.40245
		Pi-Alkyl	TZD core (ligand)	Arg 288 (side chain)	4.94846
		Pi-Alkyl	TZD core (ligand)	Ile 341 (side chain)	4.27639
		Pi-Alkyl	2-OMe (Aryl; ligand)	Leu 330 (side chain)	4.87080
		Pi-Alkyl	2-OMe (Aryl; ligand)	Val 339 (side chain)	5.01580
		Pi-Alkyl	N-Aryl (ligand)	Ile 281 (side chain)	4.99136
		Pi-Alkyl	N-Aryl (ligand)	Met 348 (side chain)	5.24193
**7b**	-7.3	PS	Leu 318 (Methyl, side chain)	TZD core (ligand)	3.63093
		PS	Leu 468 (Methyl, side chain)	N-Aryl (ligand)	3.63461
		PS	Leu 468 (Methyl, side chain)	N-Aryl (ligand)	3.96613
		Alkyl	2-Cl-Aryl (ligand)	Val 315 (side chain)	5.14187
		Alkyl	2-Cl-Aryl (ligand)	Pro 467 (side chain)	4.68961
		Alkyl	Methyl (N-Aryl, ligand)	Leu 468 (side chain)	4.69788
		Pi-Alkyl	2-Cl-Aryl (ligand)	Leu 311 (side chain)	5.49394
		Pi-Alkyl	2-Cl-Aryl (ligand)	Pro 467 (side chain)	5.44119
		Pi-Alkyl	N-Aryl (ligand)	Pro 467 (side chain)	5.10571
**7c**	-7.1	HB	Asn 308 (NH_2_)	2-NO_2_-Aryl (ligand)	3.25728
		PS	Pro 304 (αCH)	2-NO_2_-Aryl (ligand)	3.77999
		Alkyl	Methyl (N-Aryl, ligand)	Ala 213 (side chain)	4.46163
**7d**	-8.5	CHB	Lys 367 (side chain CH_2_NH_2_)	C = O (2nd position of TZD; ligand)	3.70277
		PDHB; PS	Cys 285 (SH)	3-OH-Aryl; TZD core (ligand)	3.99461
		Pi-Sigma	Leu 330 (side chain)	TZD core (ligand)	3.84019
		Pi-Sigma	Ile 341 (side chain)	3-OH-Aryl (ligand)	3.50391
		PS	Cys 285 (SH)	N-Aryl (ligand)	5.46855
		PS	Cys 285 (SH)	3-OH-Aryl (ligand)	5.82541
		PPT	His 449 (Imidazole ring)	N-Aryl (ligand)	4.96478
		APS	Cys 285; Gln 286 (CONH)	N-Aryl (ligand)	5.20028
		Pi-Alkyl	3-OH-Aryl (ligand)	Arg 288 (side chain)	3.94502
		Pi-Alkyl	Phe 282 (Aryl)	Methyl (N-Aryl, ligand)	4.90419
		Pi-Alkyl	His 449 (Imidazole ring)	Methyl (N-Aryl, ligand)	4.80268
**7e**	-8.9	PDHB; PS	Cys 285 (SH)	N-Aryl; TZD core (ligand)	3.68179
		Pi-Sigma	Arg 288 (side chain)	N-Aryl (ligand)	3.55725
		Pi-Sigma	Ile 341 (side chain)	Methyl (N-Aryl, ligand)	3.77542
		PS	Cys 285 (SH)	N-Aryl (ligand)	5.46193
		PPT	His 449 (Imidazole ring)	4-Cl-Aryl (ligand)	4.67448
		APT	Cys 285; Gln 286 (CONH)	4-Cl-Aryl (ligand)	4.81318
		Alkyl	4-Cl-Aryl (ligand)	Leu 469 (side chain)	4.17882
		Alkyl	Methyl (N-Aryl, ligand)	Leu 270 (side chain)	4.88063
		Alkyl	Methyl (N-Aryl, ligand)	Arg 288 (side chain)	4.06932
		Alkyl	Methyl (N-Aryl, ligand)	Ile 341 (side chain)	4.55730
		Pi-Alkyl	TZD core (ligand)	Leu 330 (side chain)	4.65177
		Pi-Alkyl	4-Cl-Aryl (ligand)	Cys 285 (side chain)	5.15119
		Pi-Alkyl	His 323 (Imidazole ring)	4-Cl-Aryl (ligand)	4.39467
		Pi-Alkyl	His 449 (Imidazole ring)	4-Cl-Aryl (ligand)	5.03461

The various types of non-covalent interactions of each molecule with the receptor was shown in **Figs 3–9**. Most of the amino acids from the protein that have interacted with the synthesized compounds are either hydrophobic or basic. Hydrophobic interactions usually occurred from alkyl/pi-alkyl to amino acids such as Leu, His, Arg etc. There are a few polar interactions (hydrogen bond, carbon hydrogen bond and Pi-Donor hydrogen bond) as well as other interactions also occurred dependent on the electronic environment (amide pi-stacked, pi-pi t stacked, pi-sigma, etc). It should be noted that binding site for protein is not similar for all synthesized molecules. All these data indicate that the core structure of our synthesized compounds is very cooperative with the protein.

## Conclusion

In conclusion, we developed a convenient method to synthesize 3-*m*-tolyl-5-arylidenthiazolidine- 2,4-dione derivatives by using morpholine as a catalyst. Molecular flexible docking studies have shown that our synthesized compounds are very active and some of them shown better binding affinity with the protein than the commercially available drugs, Epalrestat. These results inspire us to study the moiety even further and test these molecules for their biological activity. To find out more potent compound we plan to do pharmacokinetics study in near future.

## Supporting information

S1 Fig UV spectrum of 3-(*m*-tolyl) thiazolidine-2, 4- dione (4). (DOCX)

S2 Fig IR spectrum of 3-(*m*-tolyl) thiazolidine-2, 4- dione (4). (DOCX)

S3 Fig ^1^H NMR spectrum of 3-(*m*-tolyl) thiazolidine-2, 4- dione (4). (DOCX)

S4 Fig ^13^C NMR spectrum of 3-(*m*-tolyl) thiazolidine-2, 4- dione (4). (DOCX)

S5 Fig DEPT-135 spectrum of 3-(*m*-tolyl) thiazolidine-2, 4- dione (4). (DOCX)

S6 Fig UV of 5-(2-Methoxybenzylidene)-3-*m*-tolyl thiazolidine-2, 4- dione (7a). (DOCX)

S7 Fig IR spectrum of 5-(2-Methoxybenzylidene)-3-*m*-tolyl thiazolidine-2, 4- dione (7a). (DOCX)

S8 Fig ^1^H NMR spectrum of 5-(2-Methoxybenzylidene)-3-*m*-tolyl thiazolidine-2, 4- dione (7a). (DOCX)

S9 Fig ^13^C NMR spectrum of 5-(2-Methoxybenzylidene)-3-*m*-tolyl thiazolidine-2, 4- dione (7a). (DOCX)

S10 Fig DEPT-135 spectrum of 5-(2-Methoxybenzylidene)-3-*m*-tolyl thiazolidine-2, 4- dione (7a). (DOCX)

S11 Fig UV spectrum of 5-(2-Chlorobenzylidene)-3-*m*-tolyl thiazolidine-2, 4- dione (7b). (DOCX)

S12 Fig IR spectrum of 5-(2-Chlorobenzylidene)-3-*m*-tolyl thiazolidine-2, 4- dione (7b). (DOCX)

S13 Fig ^1^H NMR spectrum of 5-(2-Chlorobenzylidene)-3-*m*-tolyl thiazolidine-2, 4- dione (7b). (DOCX)

S14 Fig ^13^C NMR spectrum of 5-(2-Chlorobenzylidene)-3-*m*-tolyl thiazolidine-2, 4- dione (7b). (DOCX)

S15 Fig DEPT-135 spectrum of 5-(2-Chlorobenzylidene)-3-*m*-tolyl thiazolidine-2, 4- dione (7b). (DOCX)

S16 Fig UV spectrum of 5-(2-Nitrobenzylidene)-3-*m*-tolyl thiazolidine-2, 4- dione (7c). (DOCX)

S17 Fig IR spectrum of 5-(2-Nitrobenzylidene)-3-*m*-tolyl thiazolidine-2, 4- dione (7c). (DOCX)

S18 Fig ^1^H NMR spectrum of 5-(2-Nitrobenzylidene)-3-*m*-tolyl thiazolidine-2, 4- dione (7c). (DOCX)

S19 Fig ^13^C NMR spectrum of 5-(2-Nitrobenzylidene)-3-*m*-tolyl thiazolidine-2, 4- dione (7c). (DOCX)

S20 Fig DEPT-135 spectrum of 5-(2-Nitrobenzylidene)-3-*m*-tolyl thiazolidine-2, 4- dione (7c). (DOCX)

S21 Fig UV spectrum of 5-(3-Hydroxybenzylidene)-3-*m*-tolyl thiazolidine-2, 4- dione (7d). (DOCX)

S22 Fig ^1^H NMR spectrum of 5-(3-Hydroxybenzylidene)-3-*m*-tolyl thiazolidine-2, 4- dione (7d). (DOCX)

S23 Fig DEPT-135 NMR spectrum of 5-(3-Hydroxybenzylidene)-3-*m*-tolyl thiazolidine-2, 4- dione (7d). (DOCX)

S24 Fig ^13^C NMR spectrum of 5-(3-Hydroxybenzylidene)-3-*m*-tolyl thiazolidine-2, 4- dione (7d). (DOCX)

S25 Fig UV spectrum of 5-(4-Chlorobenzylidene)-3-*m*-tolyl thiazolidine-2, 4- dione (7e). (DOCX)

S26 Fig IR spectrum of 5-(4-Chlorobenzylidene)-3-*m*-tolyl thiazolidine-2, 4- dione (7e). (DOCX)

S27 Fig ^1^H NMR spectrum of 5-(4-Chlorobenzylidene)-3-*m*-tolyl thiazolidine-2, 4- dione (7e). (DOCX)

S28 Fig ^13^C NMR spectrum of 5-(4-Chlorobenzylidene)-3-*m*-tolyl thiazolidine-2, 4- dione (7e). (DOCX)

S29 Fig DEPT-135 spectrum of 5-(4-Chlorobenzylidene)-3-*m*-tolyl thiazolidine-2, 4- dione (7e). (DOCX)

S30 Fig Mechanism of the 3-aryl thiazolidine-2,4-dione synthesis (4). (DOCX)

S31 Fig Chemical structures of molecules optimized with DFT-B3LYP (6-31G, d) level of theory. (DOCX)

S32 Fig HOMO and LUMO structures of the optimized molecules. (DOCX)

S33 Fig (TIF)
