# Supplementary figures and images for: A convenient approach to synthesize substituted 5-Arylidene-3-m-tolyl thiazolidine-2, 4-diones by using morpholine as a catalyst and its theoretical study
Source: PLoS One. 2021 Mar 4;16(3):e0247619. doi: 10.1371/journal.pone.0247619 (PMC7932548; doi:10.1371/journal.pone.0247619)

**S1 Fig: UV Spectrum of 3-*m*-tolyl tiazolidine-2, 4- dione (4)**


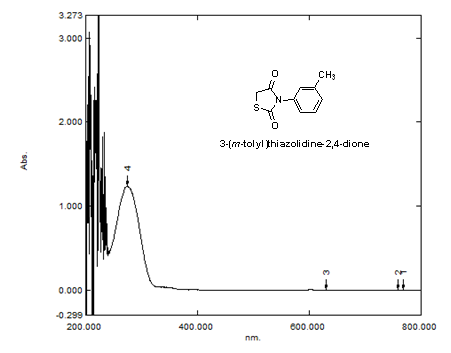

Supplement: S1 Fig — (DOCX) [file pone.0247619.s001.docx]

**S2 Fig: IR Spectrum of 3-*m*-tolyl tiazolidine-2, 4- dione (4)**


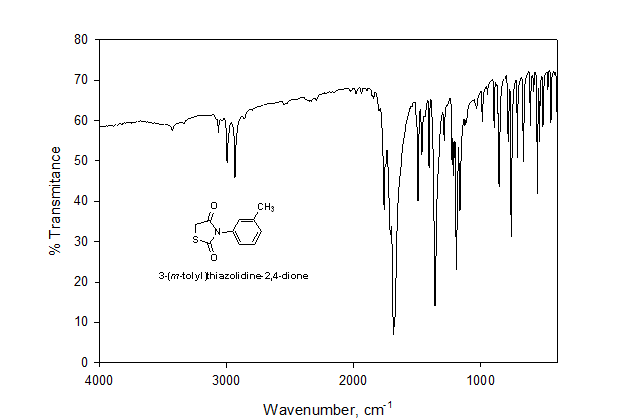

Supplement: S2 Fig — (DOCX) [file pone.0247619.s002.docx]

**S5 Fig: DEPT-135 Spectrum of 3-*m*-tolyl tiazolidine-2, 4- dione (4)**

**
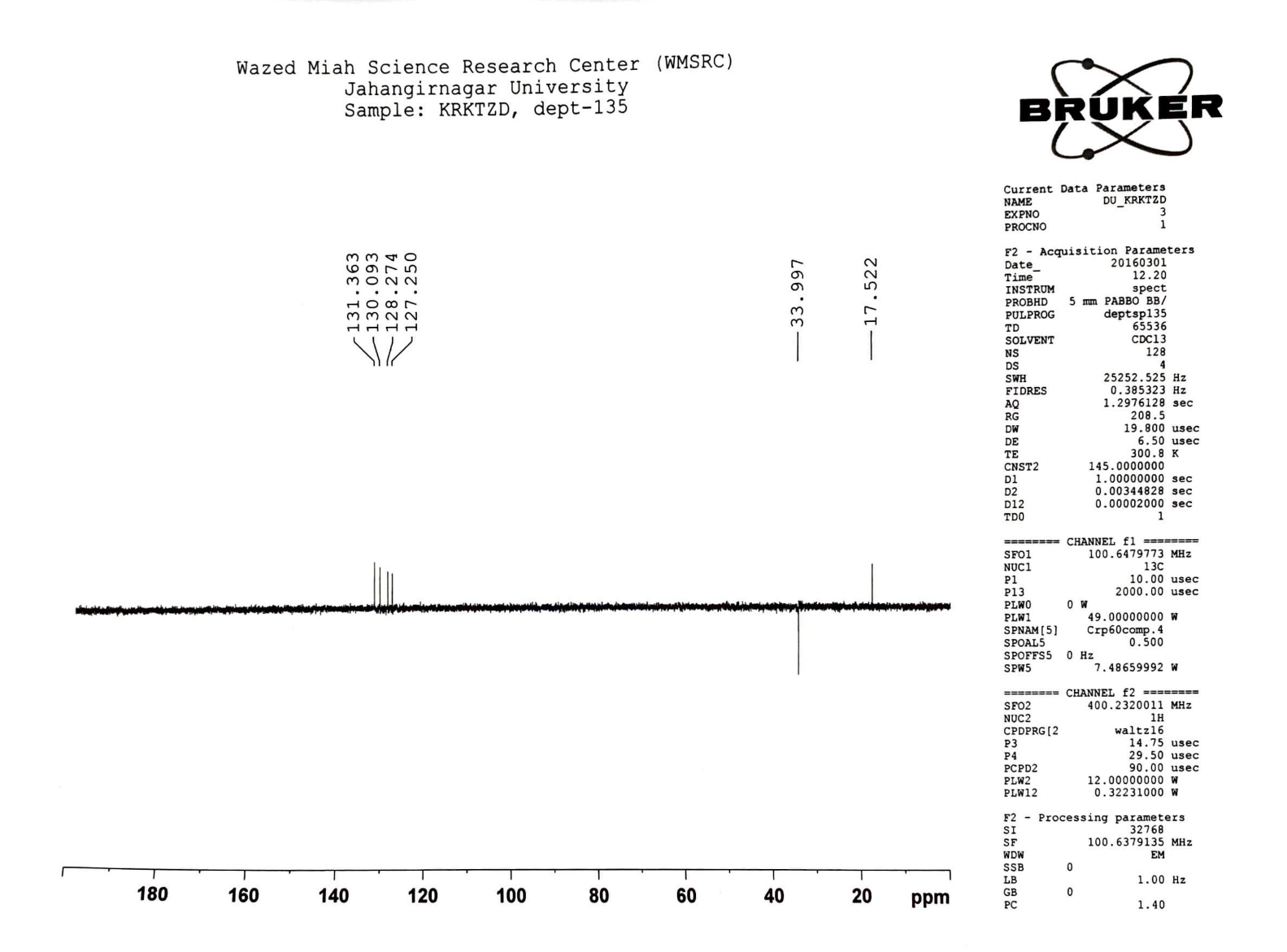
**

Supplement: S5 Fig — (DOCX) [file pone.0247619.s005.docx]

**S6 Fig: UV Spectrum of 5-(2-Methoxybenzylidene)-3-*m*-tolyl thiazolidine-2, 4- dione (7a)**


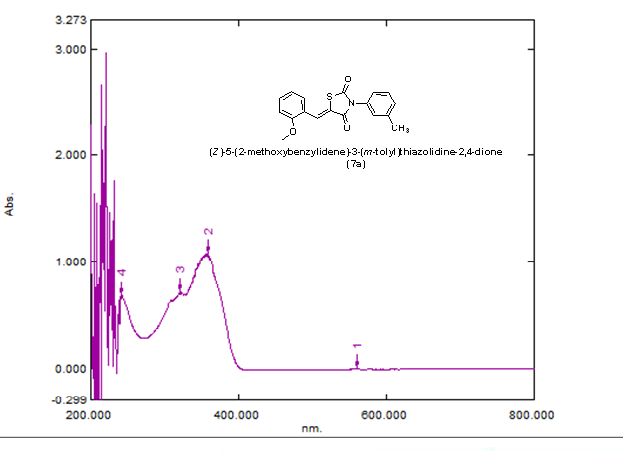

Supplement: S6 Fig — (DOCX) [file pone.0247619.s006.docx]

**S7 Fig: IR Spectrum of 5-(2-Methoxybenzylidene)-3-*m*-tolyl thiazolidine-2, 4- dione (7a)**

**
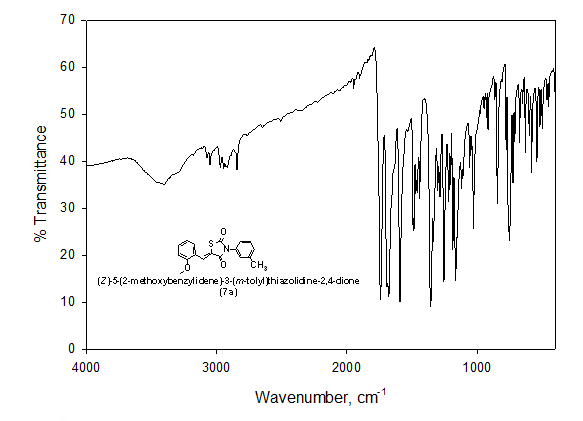
V**

Supplement: S7 Fig — (DOCX) [file pone.0247619.s007.docx]

**S8 Fig: 1H-NMR Spectrum of 5-(2-Methoxybenzylidene)-3-*m*-tolyl thiazolidine-2, 4- dione (7a)**


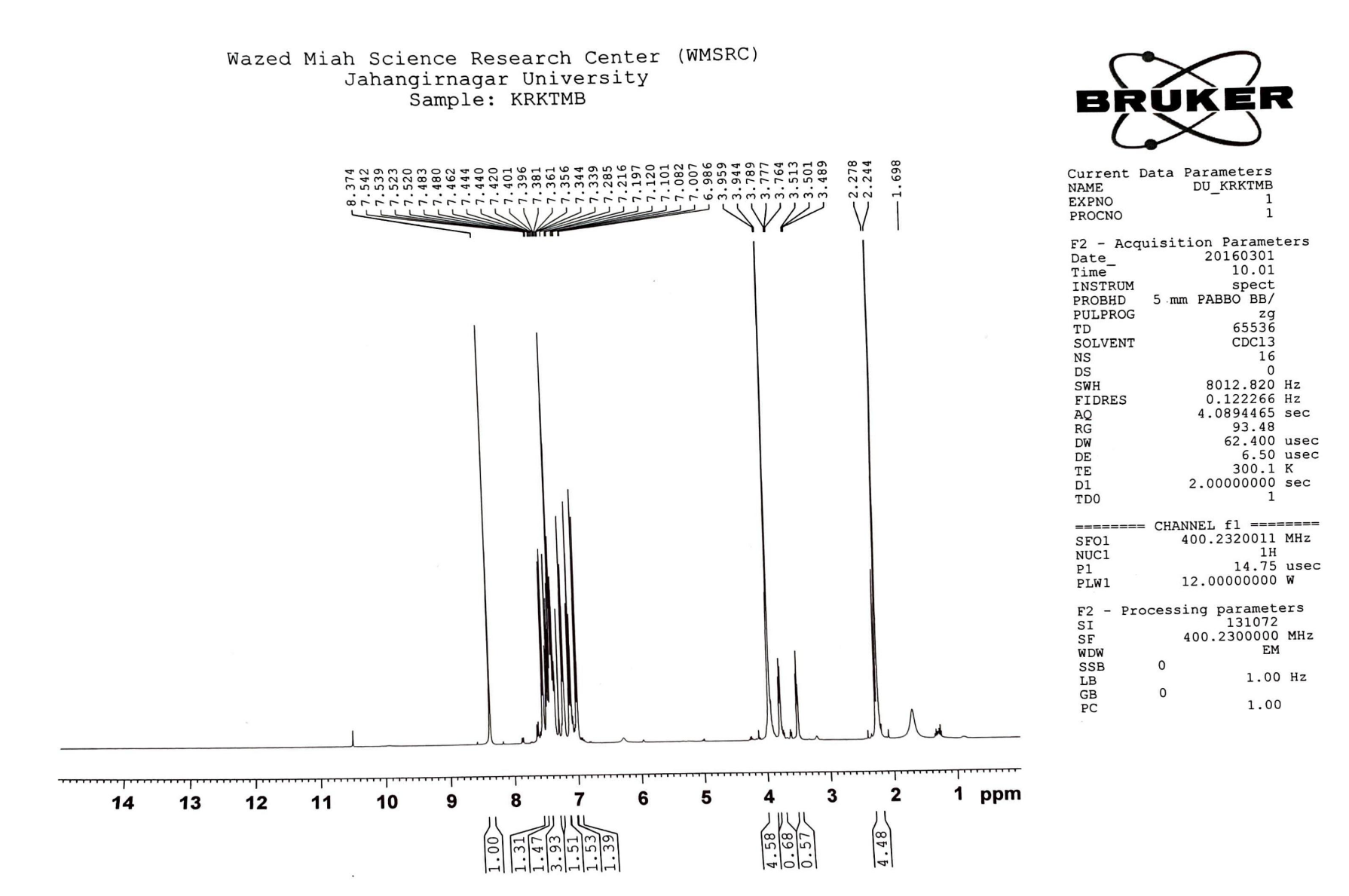

Supplement: S8 Fig — (DOCX) [file pone.0247619.s008.docx]

**S9 Fig: 13C-NMR Spectrum of 5-(2-Methoxybenzylidene)-3-*m*-tolyl thiazolidine-2, 4- dione (7a)**

**
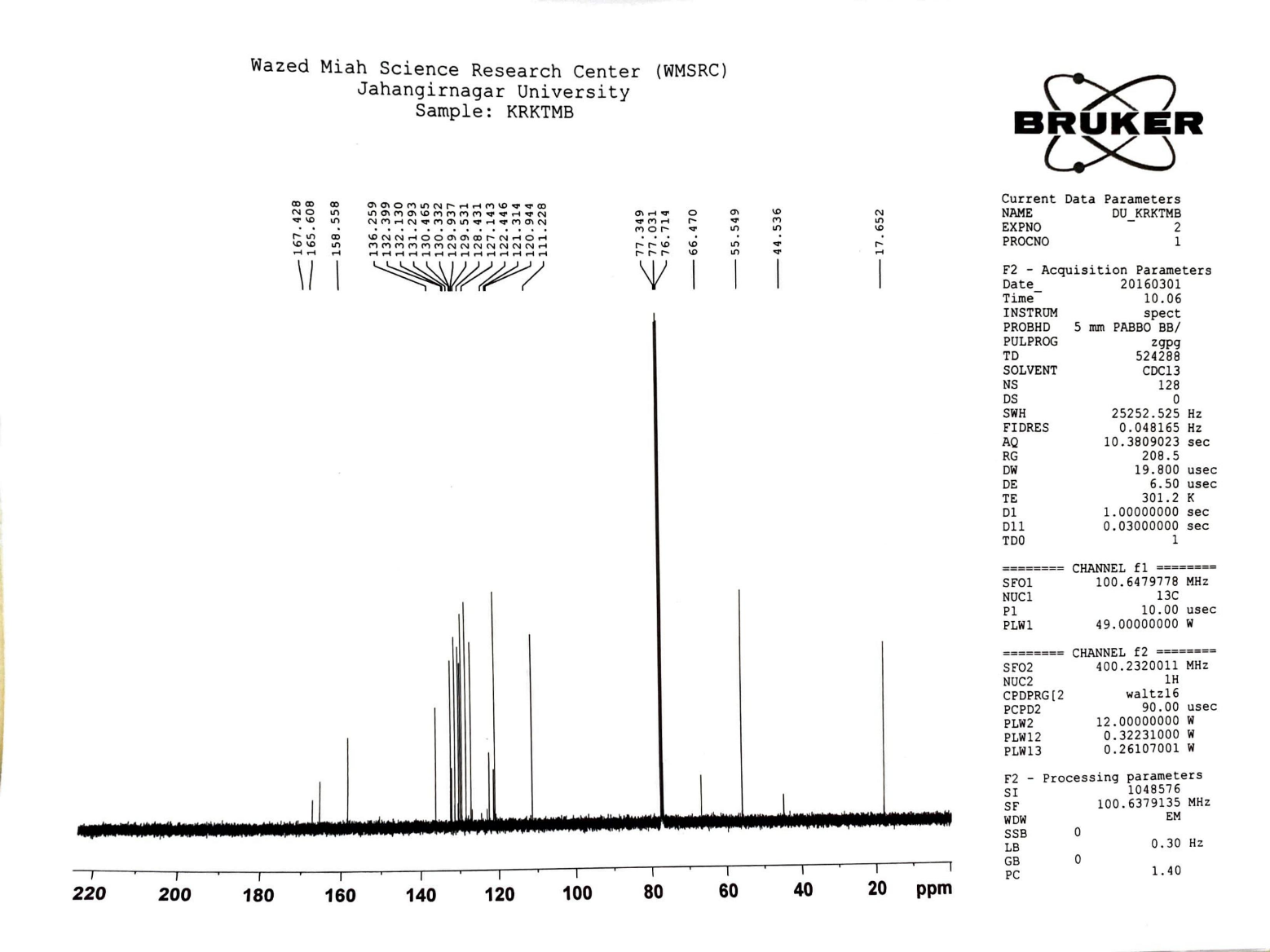
**

Supplement: S9 Fig — (DOCX) [file pone.0247619.s009.docx]

**S10 Fig: DEPT-135 Spectrum of 5-(2-Methoxybenzylidene)-3-*m*-tolyl thiazolidine-2, 4- dione (7a)**

**
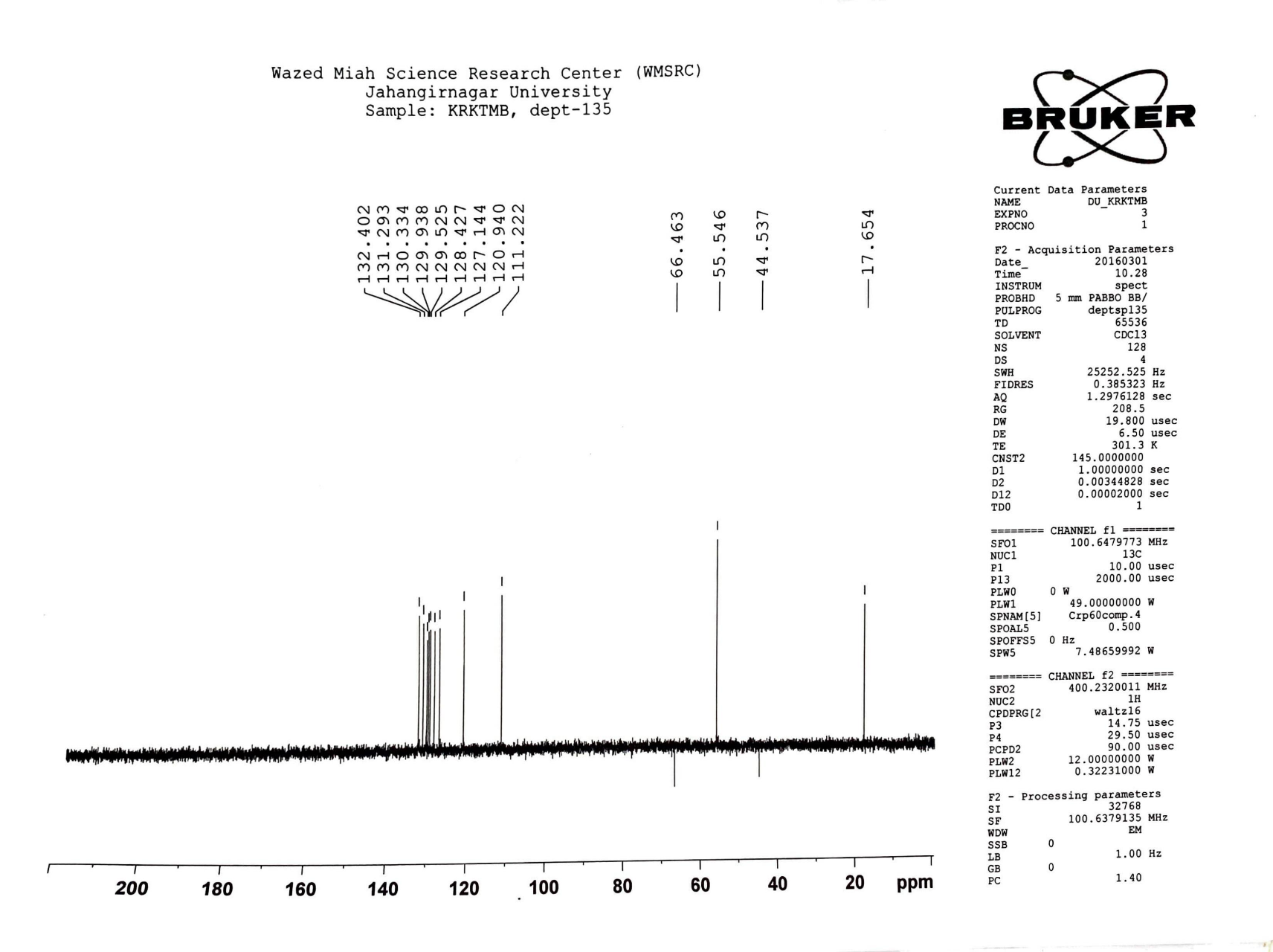
**

Supplement: S10 Fig — (DOCX) [file pone.0247619.s010.docx]

**S11 Fig: UV Spectrum of 5-(2-Chlorobenzylidene)-3-*m*-tolyl thiazolidine-2, 4- dione (7b)**


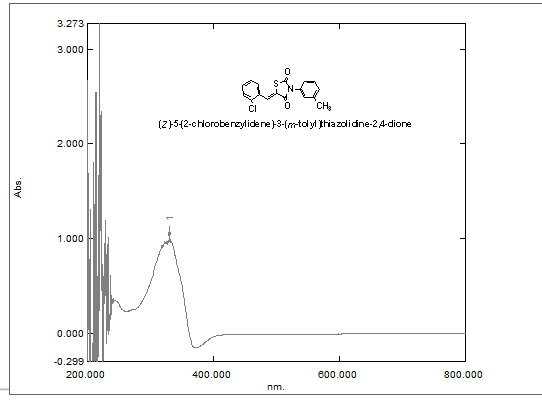

Supplement: S11 Fig — (DOCX) [file pone.0247619.s011.docx]

**S12 Fig: IR Spectrum of 5-(2-Chlorobenzylidene)-3-*m*-tolyl thiazolidine-2, 4- dione (7b)
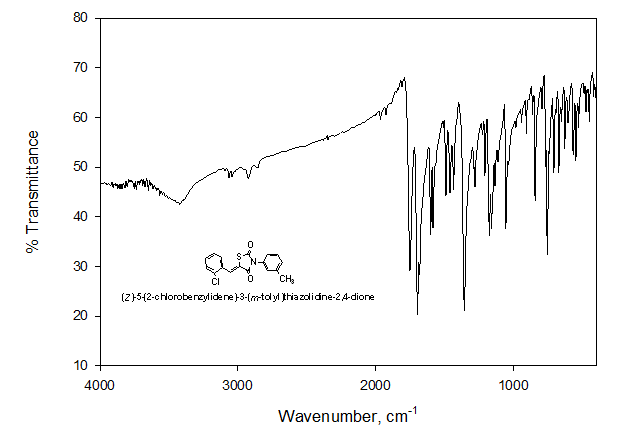
**

Supplement: S12 Fig — (DOCX) [file pone.0247619.s012.docx]

**S13 Fig: 1H-NMR Spectrum of 5-(2-Chlorobenzylidene)-3-*m*-tolyl thiazolidine-2, 4- dione (7b)**


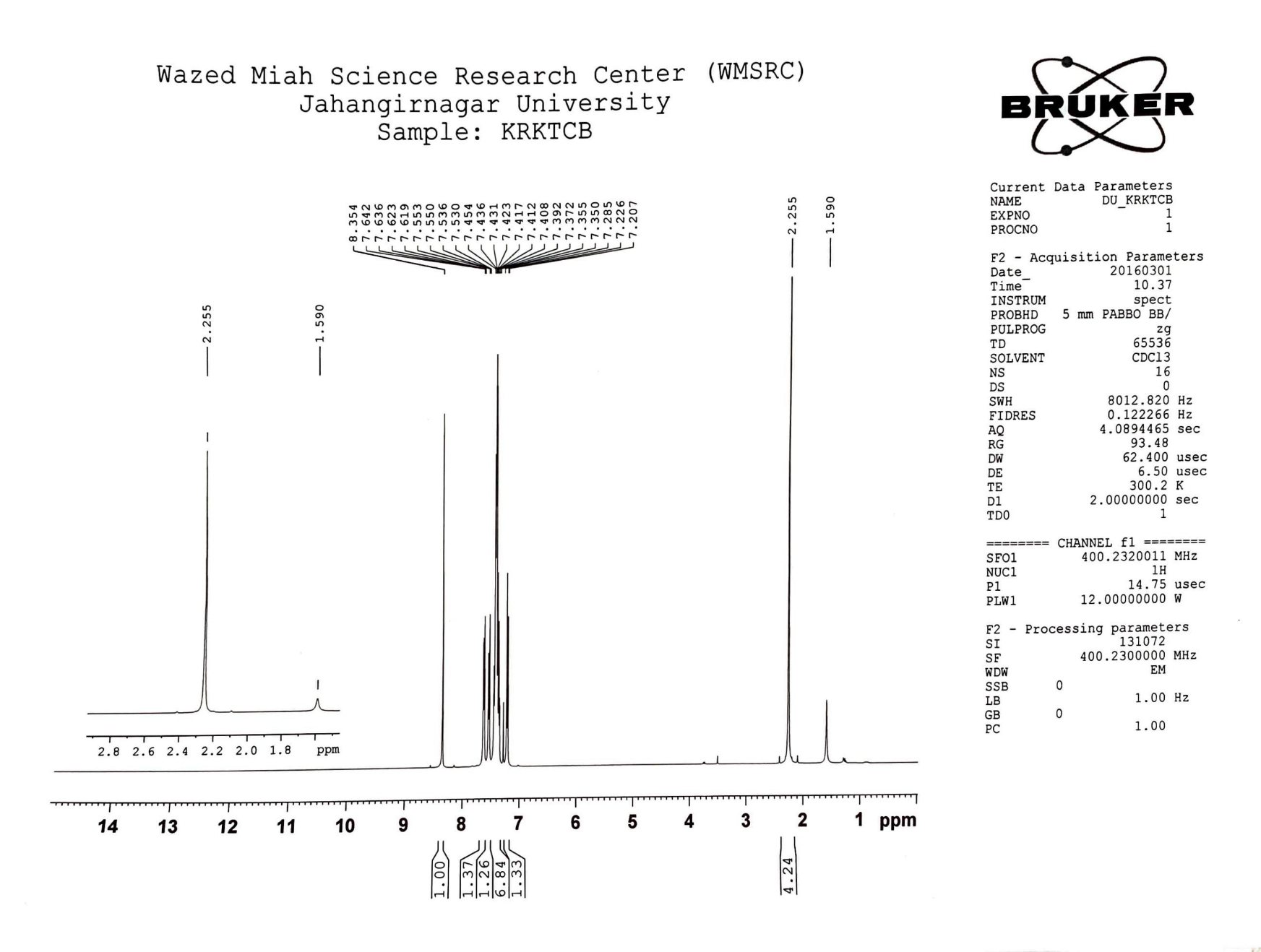

Supplement: S13 Fig — (DOCX) [file pone.0247619.s013.docx]

**S14 Fig: 1H-NMR Spectrum of 5-(2-Chlorobenzylidene)-3-*m*-tolyl thiazolidine-2, 4- dione (7b)**


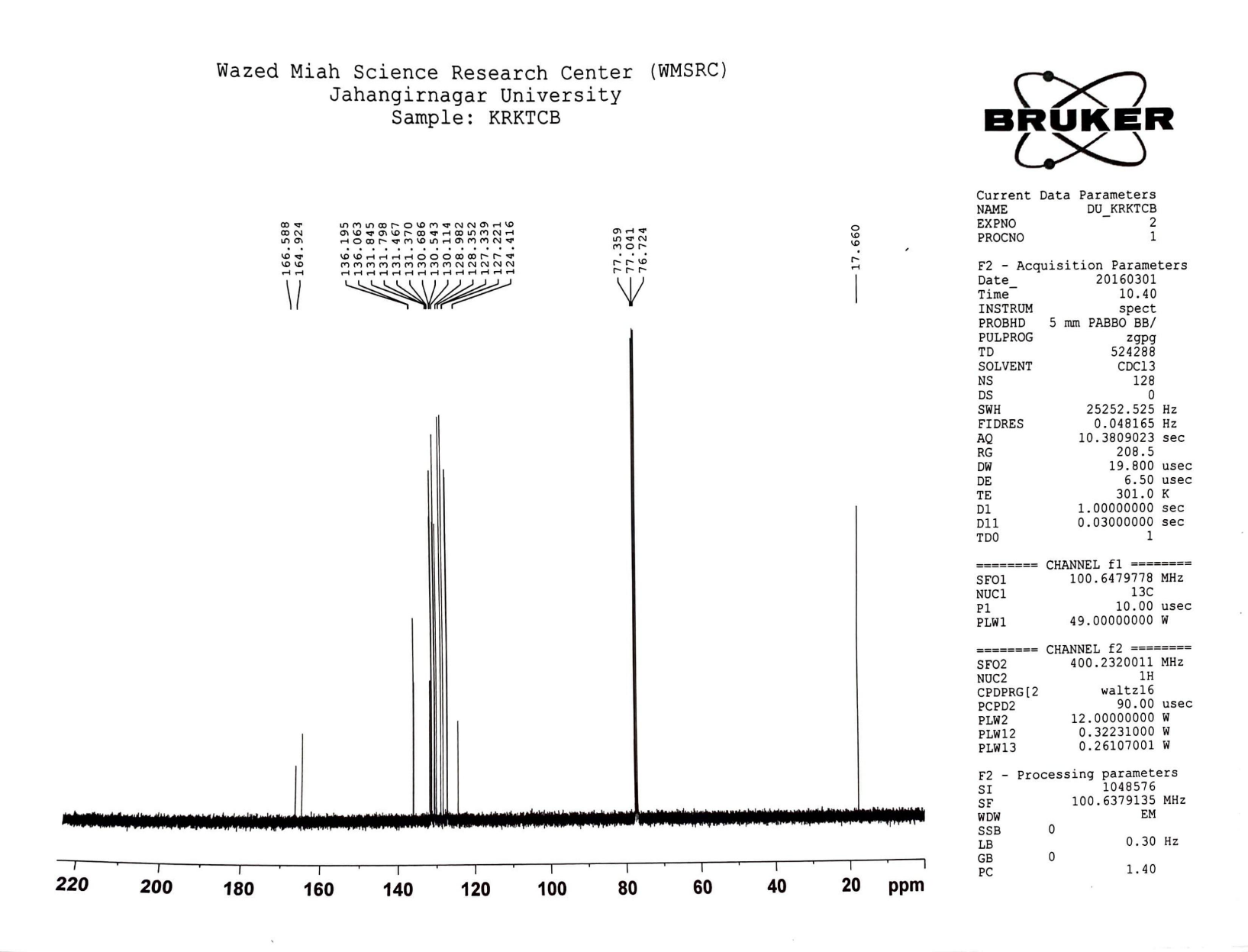

Supplement: S14 Fig — (DOCX) [file pone.0247619.s014.docx]

**S15 Fig: 1H-NMR Spectrum of 5-(2-Chlorobenzylidene)-3-*m*-tolyl thiazolidine-2, 4- dione (7b)**


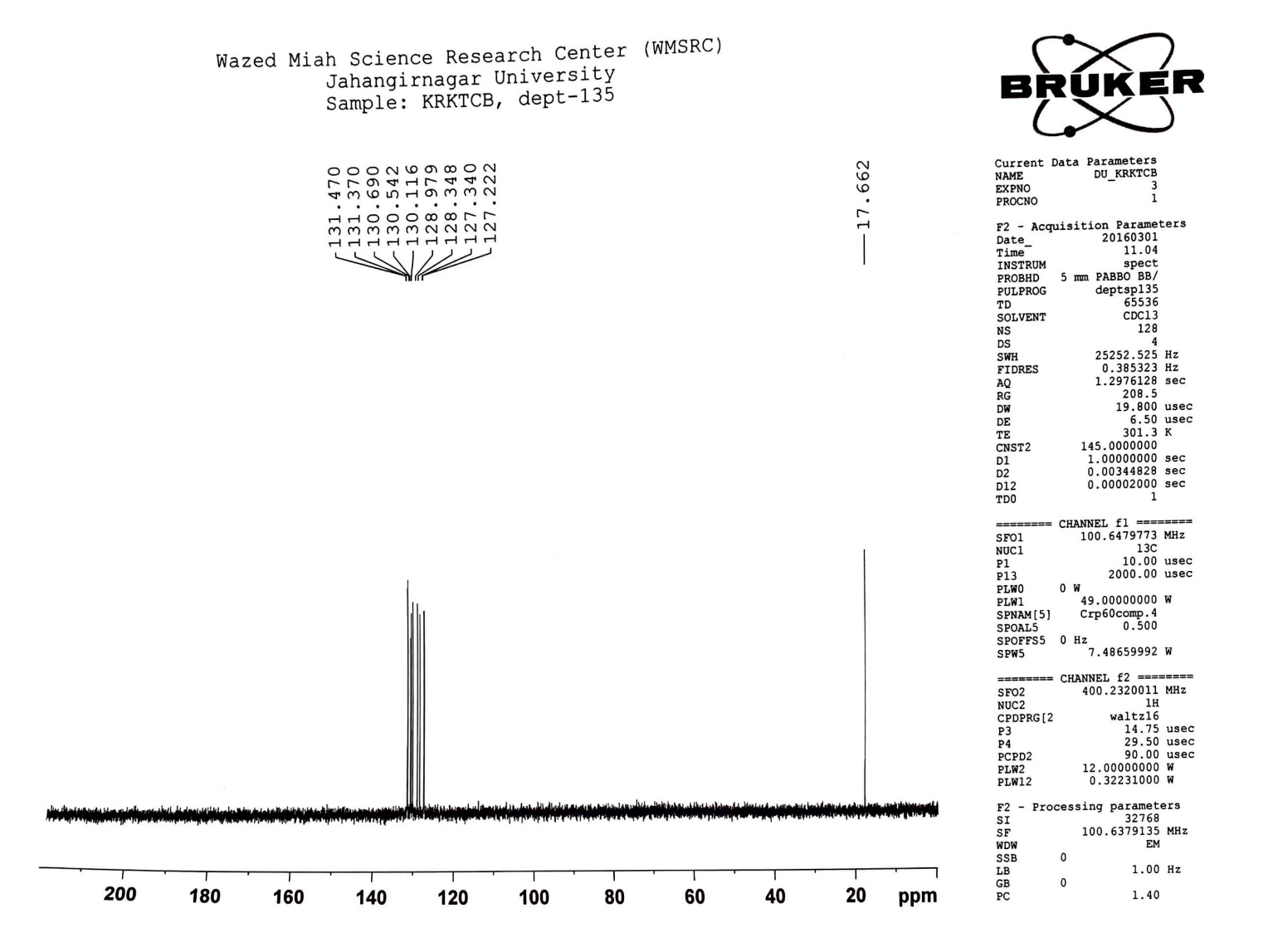

Supplement: S15 Fig — (DOCX) [file pone.0247619.s015.docx]

**S16 Fig: UV Spectrum of 5-(2-Nitrobenzylidene)-3-*m*-tolyl thiazolidine-2, 4- dione (7c)**

**
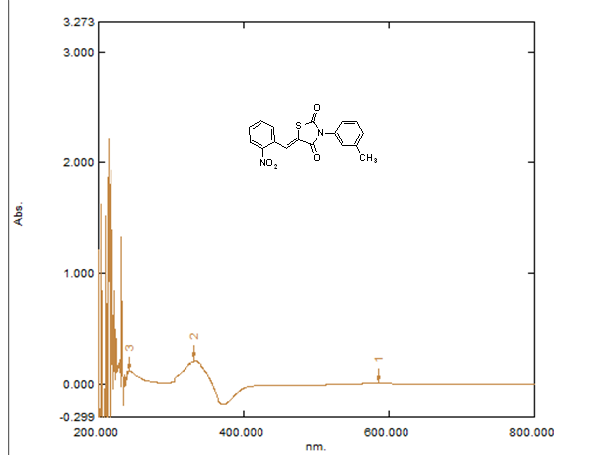
**

Supplement: S16 Fig — (DOCX) [file pone.0247619.s016.docx]

**S17 Fig: IR Spectrum of 5-(2-Nitrobenzylidene)-3-*m*-tolyl thiazolidine-2, 4- dione (7c)**


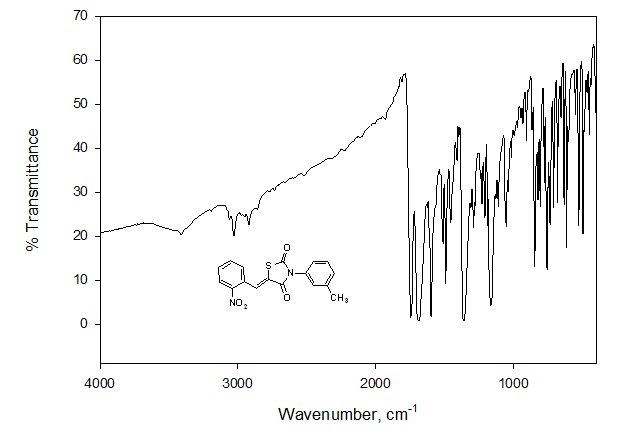

Supplement: S17 Fig — (DOCX) [file pone.0247619.s017.docx]

**S18 Fig: 1H-NMR Spectrum of 5-(2-Nitrobenzylidene)-3-*m*-tolyl thiazolidine-2, 4- dione (7c)
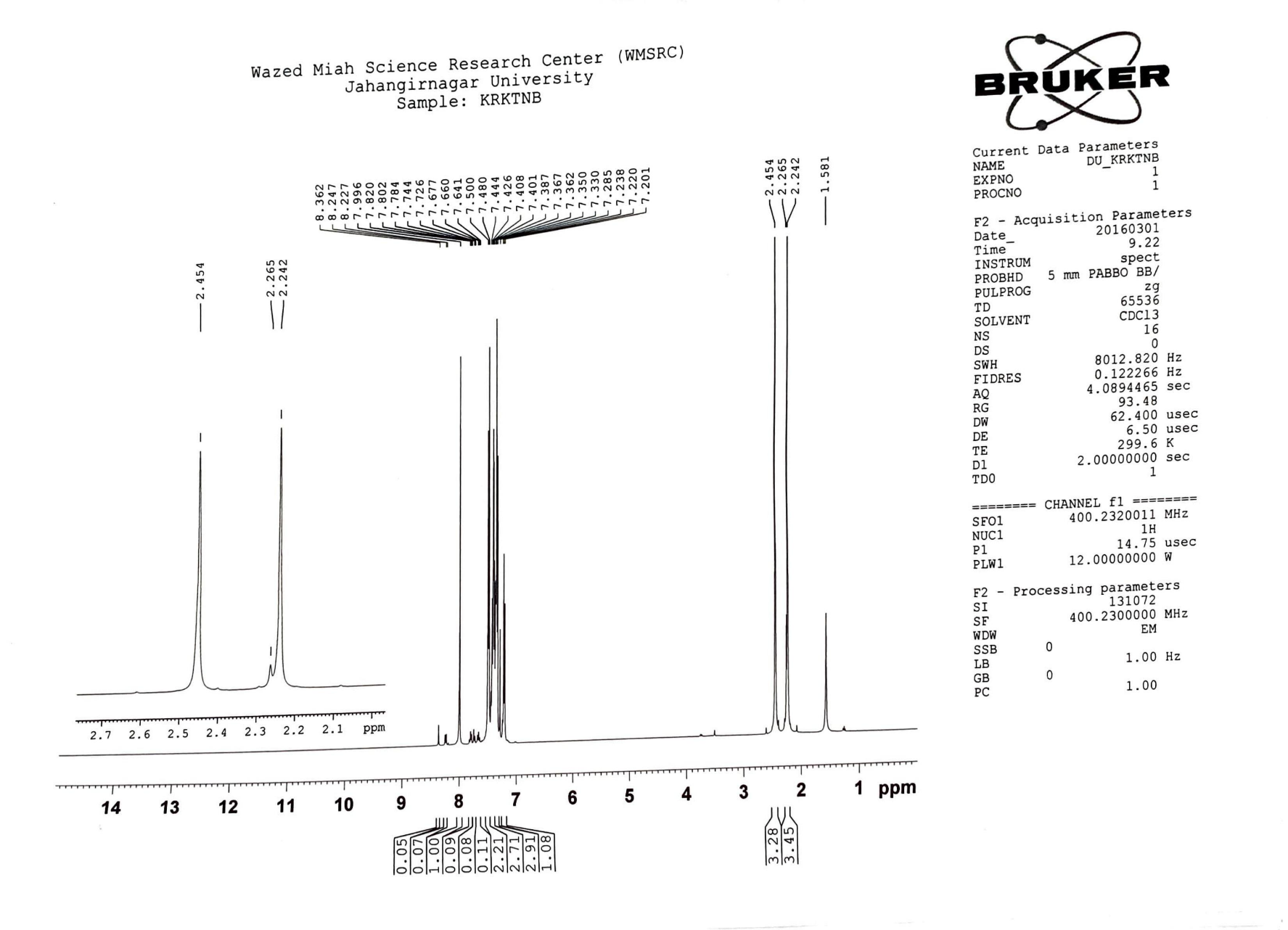
**

Supplement: S18 Fig — (DOCX) [file pone.0247619.s018.docx]

**S19 Fig: 13C-NMR Spectrum of 5-(2-Nitrobenzylidene)-3-*m*-tolyl thiazolidine-2, 4- dione (7c)**

**
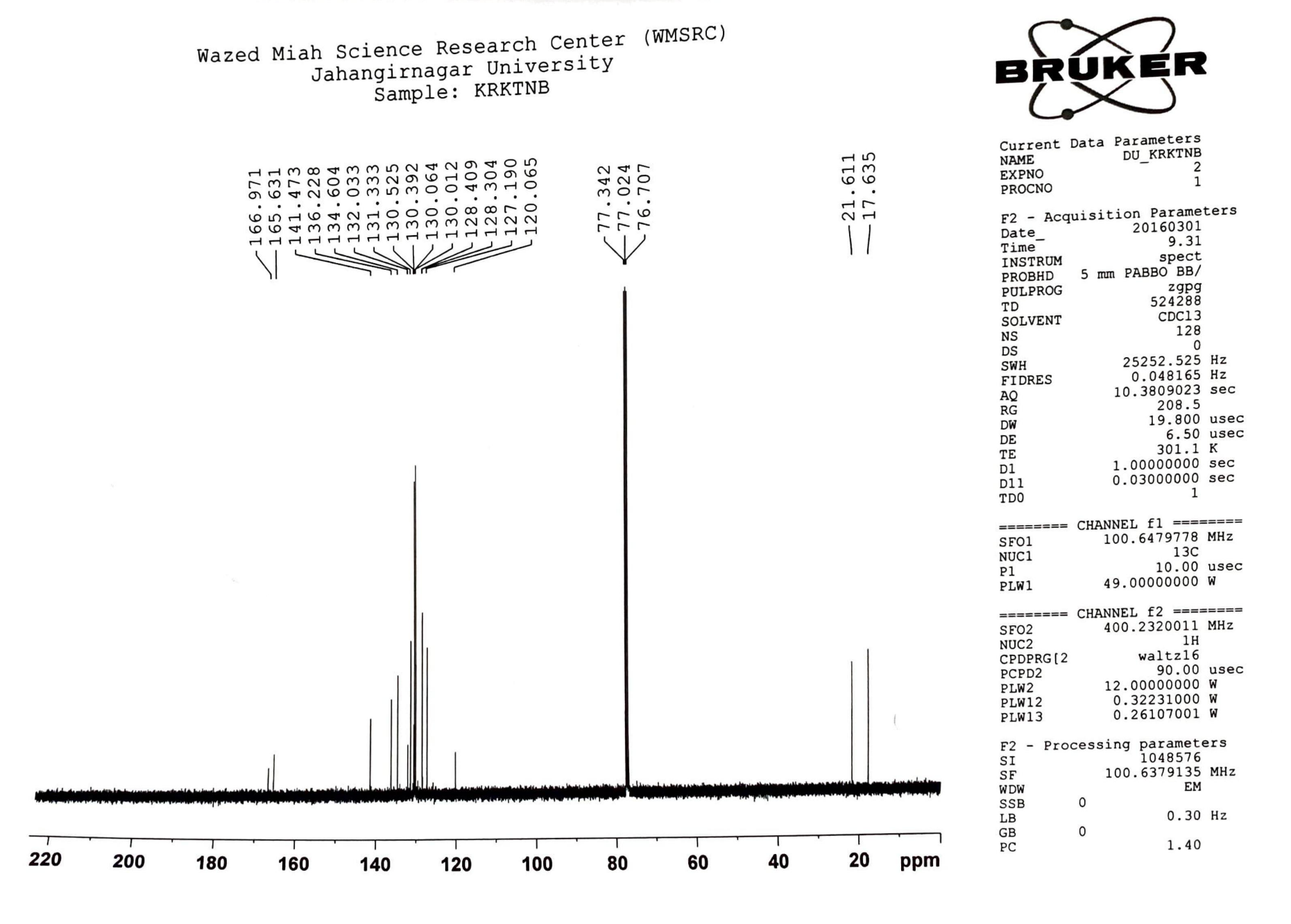
**

Supplement: S19 Fig — (DOCX) [file pone.0247619.s019.docx]

**S20 Fig: DEPT-135 Spectrum of 5-(2-Nitrobenzylidene)-3-*m*-tolyl thiazolidine-2, 4- dione (7c)**


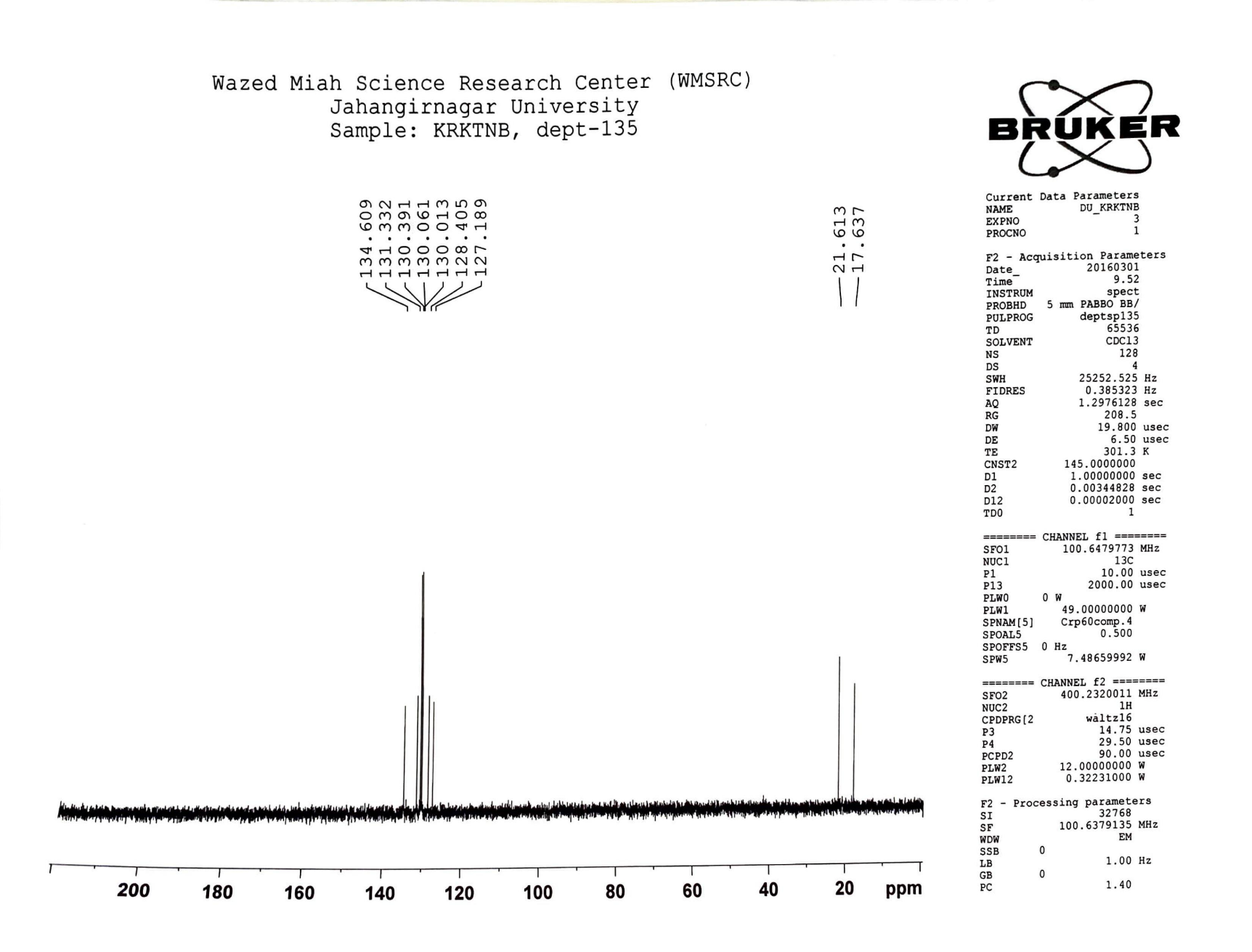

Supplement: S20 Fig — (DOCX) [file pone.0247619.s020.docx]

**S21 Fig: UV spectrum of 5-(3-Hydroxybenzylidene)-3-*m*-tolyl thiazolidine-2, 4- dione (7d)**


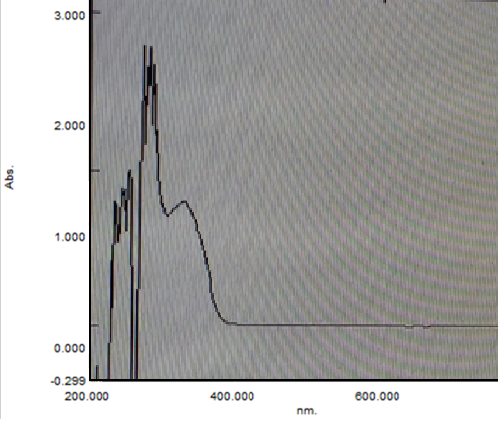

Supplement: S21 Fig — (DOCX) [file pone.0247619.s021.docx]

**S22 Fig: 1H-NMR spectrum of 5-(3-Hydroxybenzylidene)-3-*m*-tolyl thiazolidine-2, 4- dione (7d)**


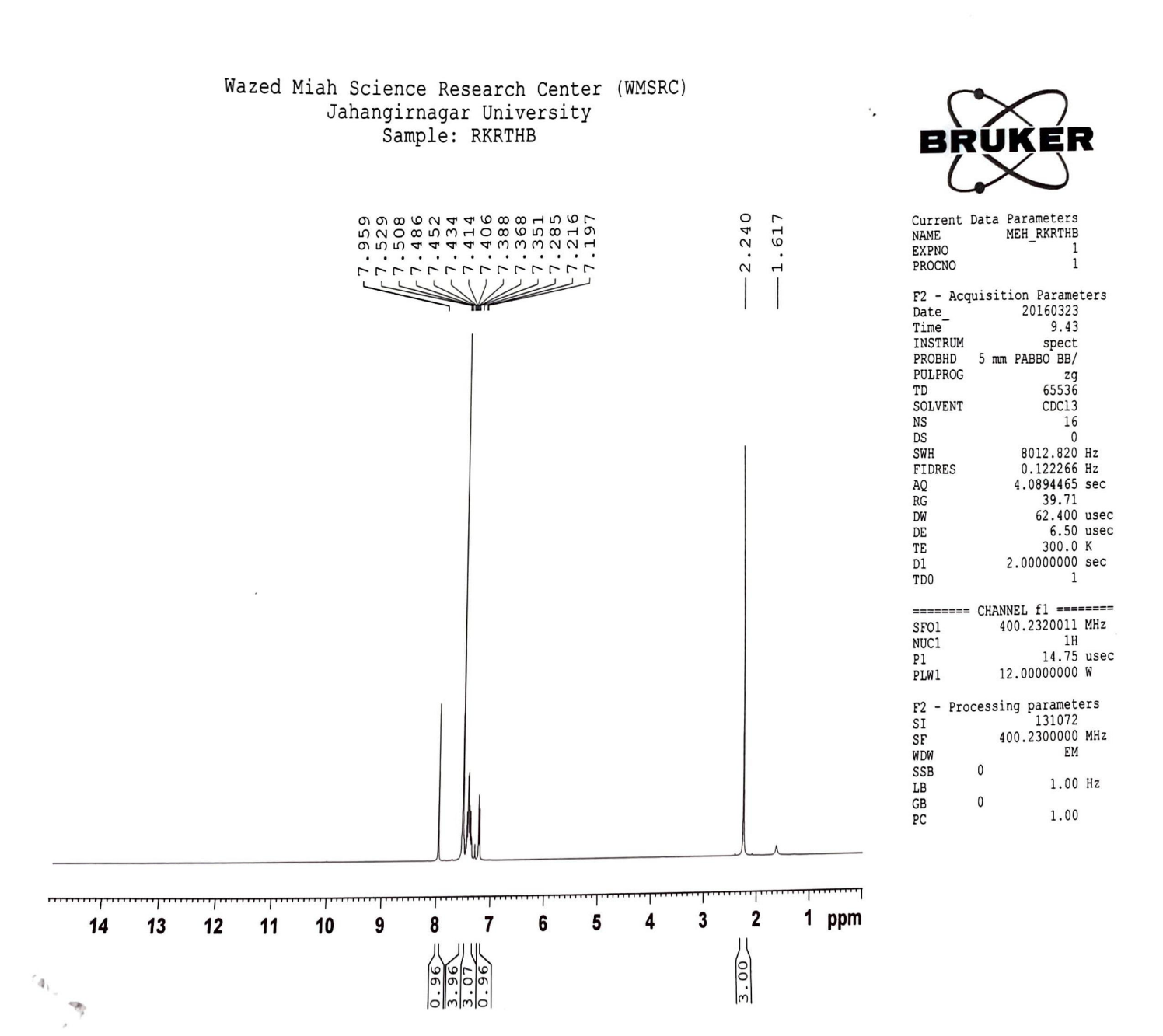

Supplement: S22 Fig — (DOCX) [file pone.0247619.s022.docx]

**S23 Fig: DEPT-135 spectrum of 5-(3-Hydroxybenzylidene)-3-*m*-tolyl thiazolidine-2, 4- dione (7d)**


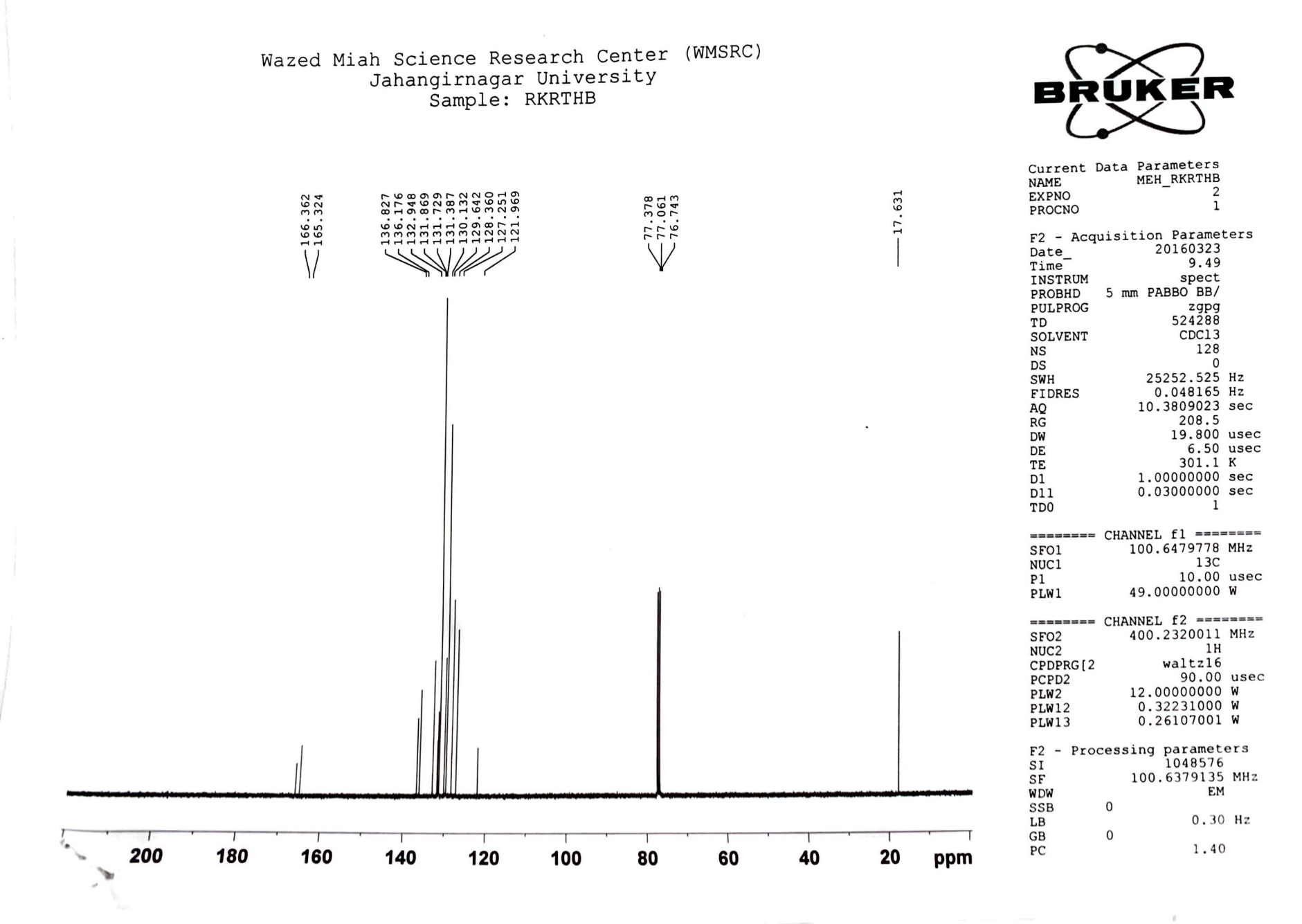

Supplement: S23 Fig — (DOCX) [file pone.0247619.s023.docx]

**S24 Fig: 13C-NMR spectrum of 5-(3-Hydroxybenzylidene)-3-*m*-tolyl thiazolidine-2, 4- dione (7d)**

**
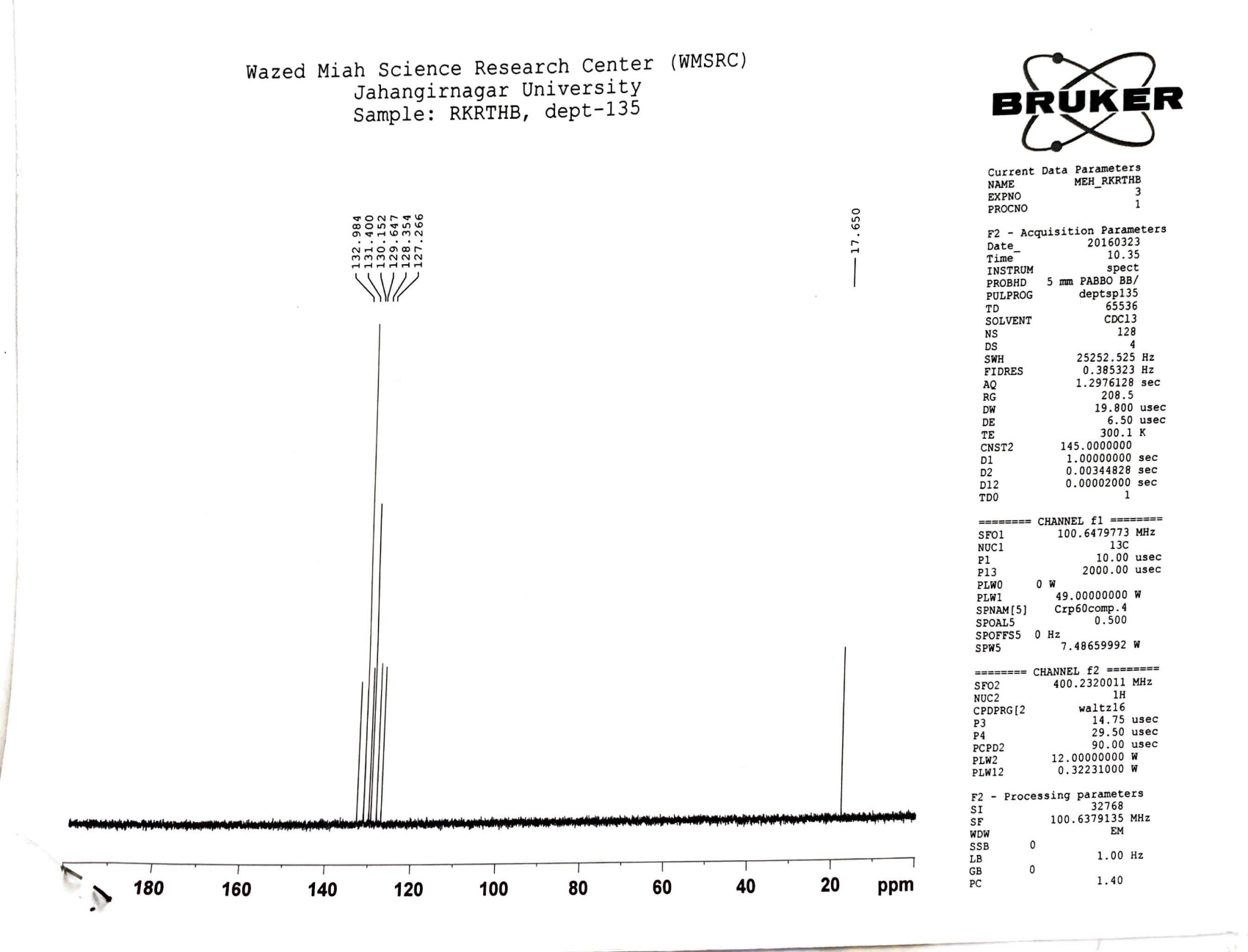
**

Supplement: S24 Fig — (DOCX) [file pone.0247619.s024.docx]

**S25 Fig: UV spectrum of 5-(4-Chlorobenzylidene)-3-*m*-tolyl thiazolidine-2, 4- dione (7e)**


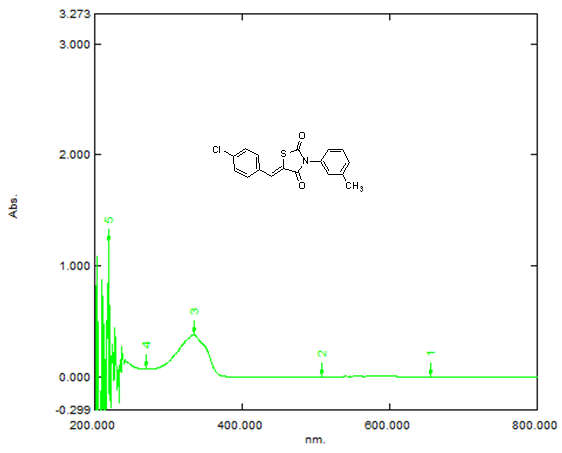

Supplement: S25 Fig — (DOCX) [file pone.0247619.s025.docx]

**S26 Fig: IR spectrum of 5-(4-Chlorobenzylidene)-3-*m*-tolyl thiazolidine-2, 4- dione (7e)**


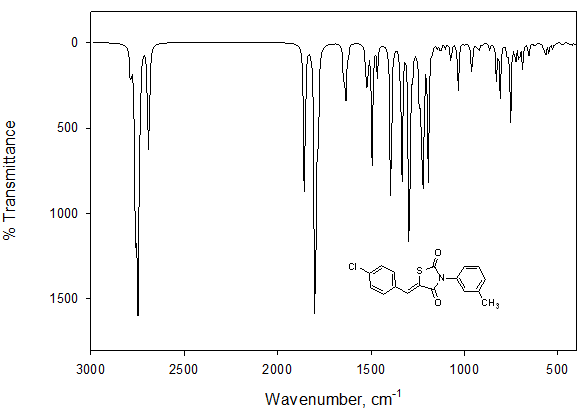

Supplement: S26 Fig — (DOCX) [file pone.0247619.s026.docx]

**S27 Fig: 1H-NMR spectrum of 5-(4-Chlorobenzylidene)-3-*m*-tolyl thiazolidine-2, 4- dione (7e)**


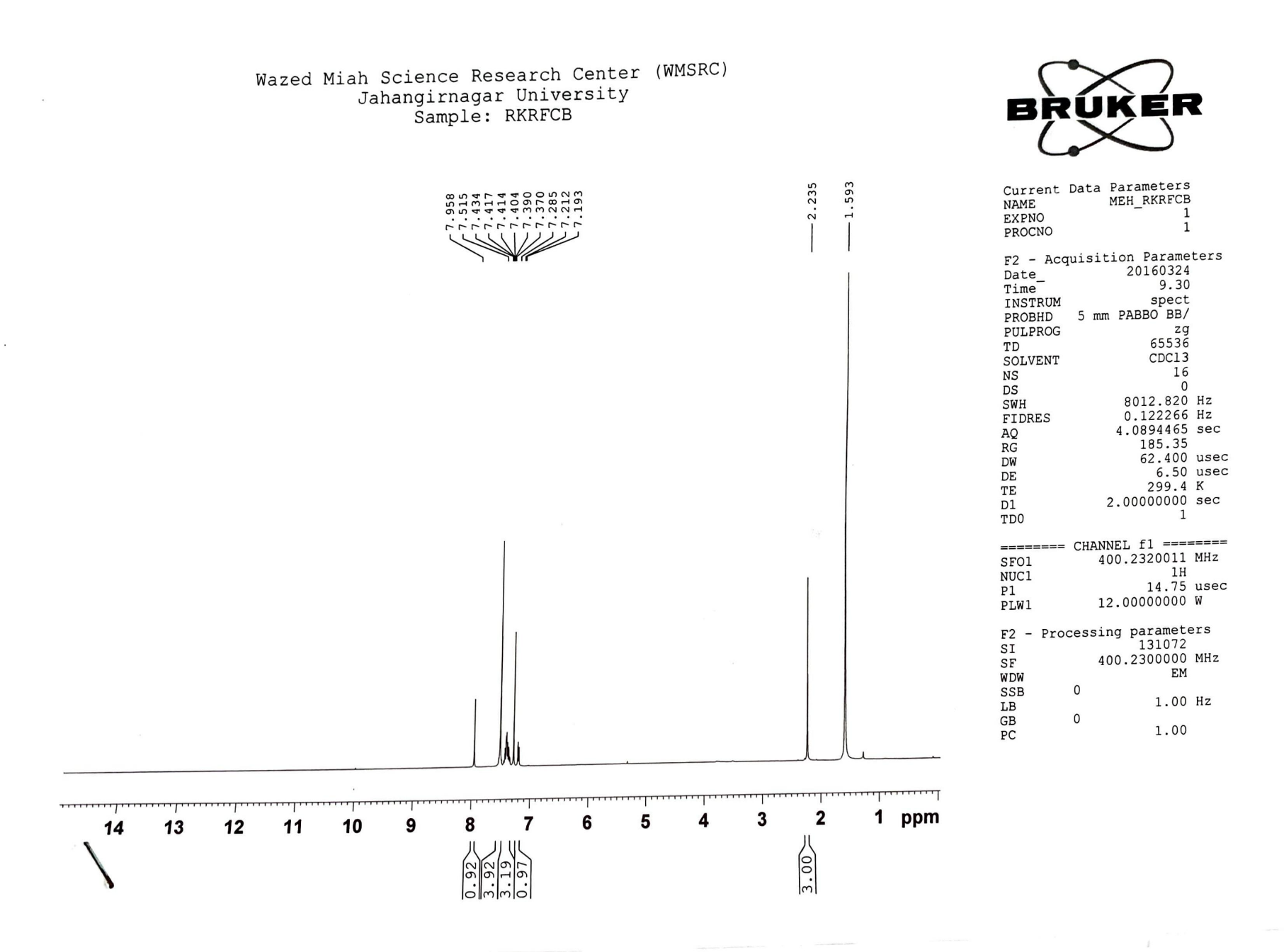

Supplement: S27 Fig — (DOCX) [file pone.0247619.s027.docx]

**S28 Fig: 13C-NMR spectrum of 5-(4-Chlorobenzylidene)-3-*m*-tolyl thiazolidine-2, 4- dione (7e)**


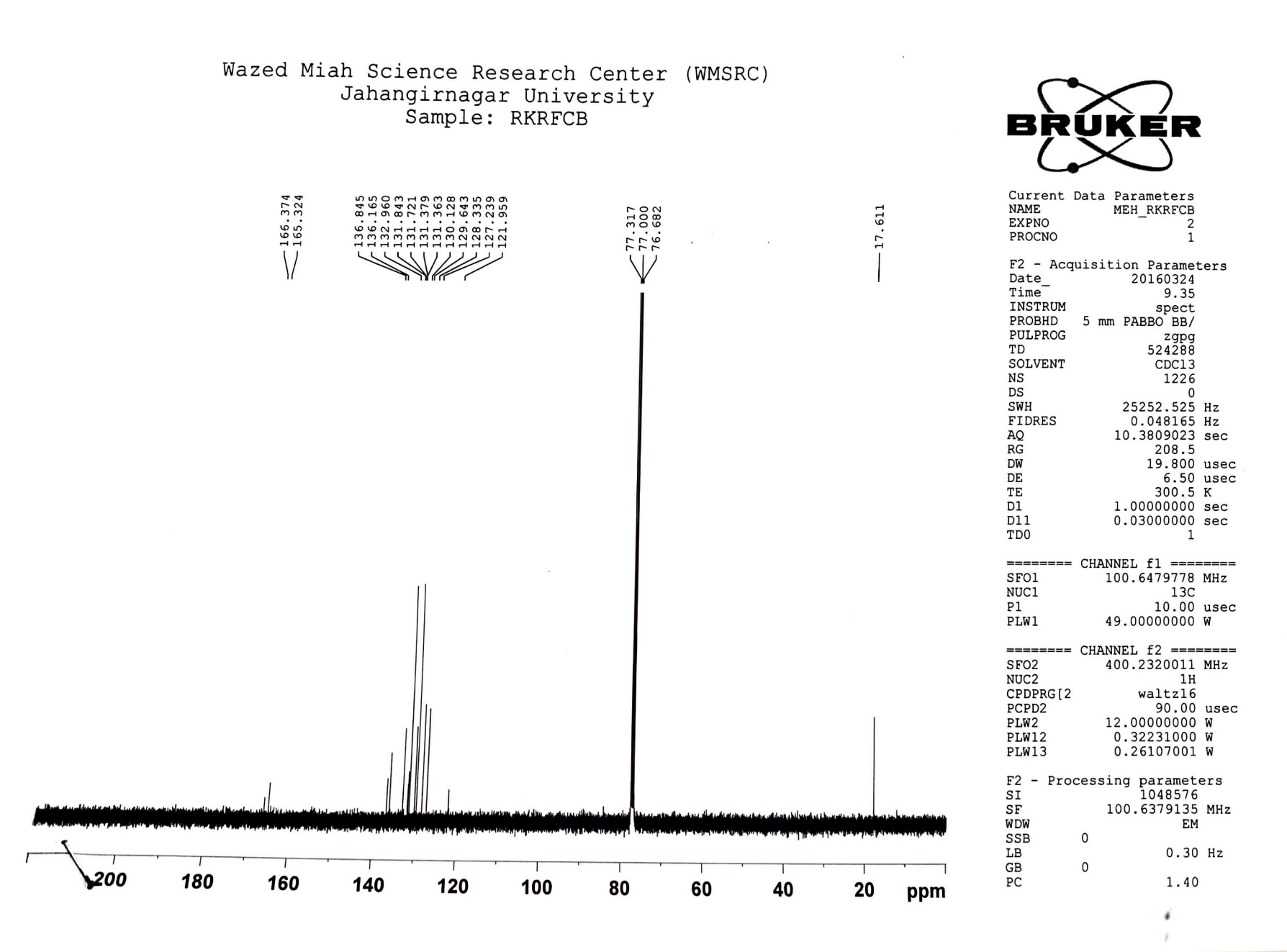

Supplement: S28 Fig — (DOCX) [file pone.0247619.s028.docx]

**S29 Fig: DEPT-135 spectrum of 5-(4-Chlorobenzylidene)-3-*m*-tolyl thiazolidine-2, 4- dione (7e**


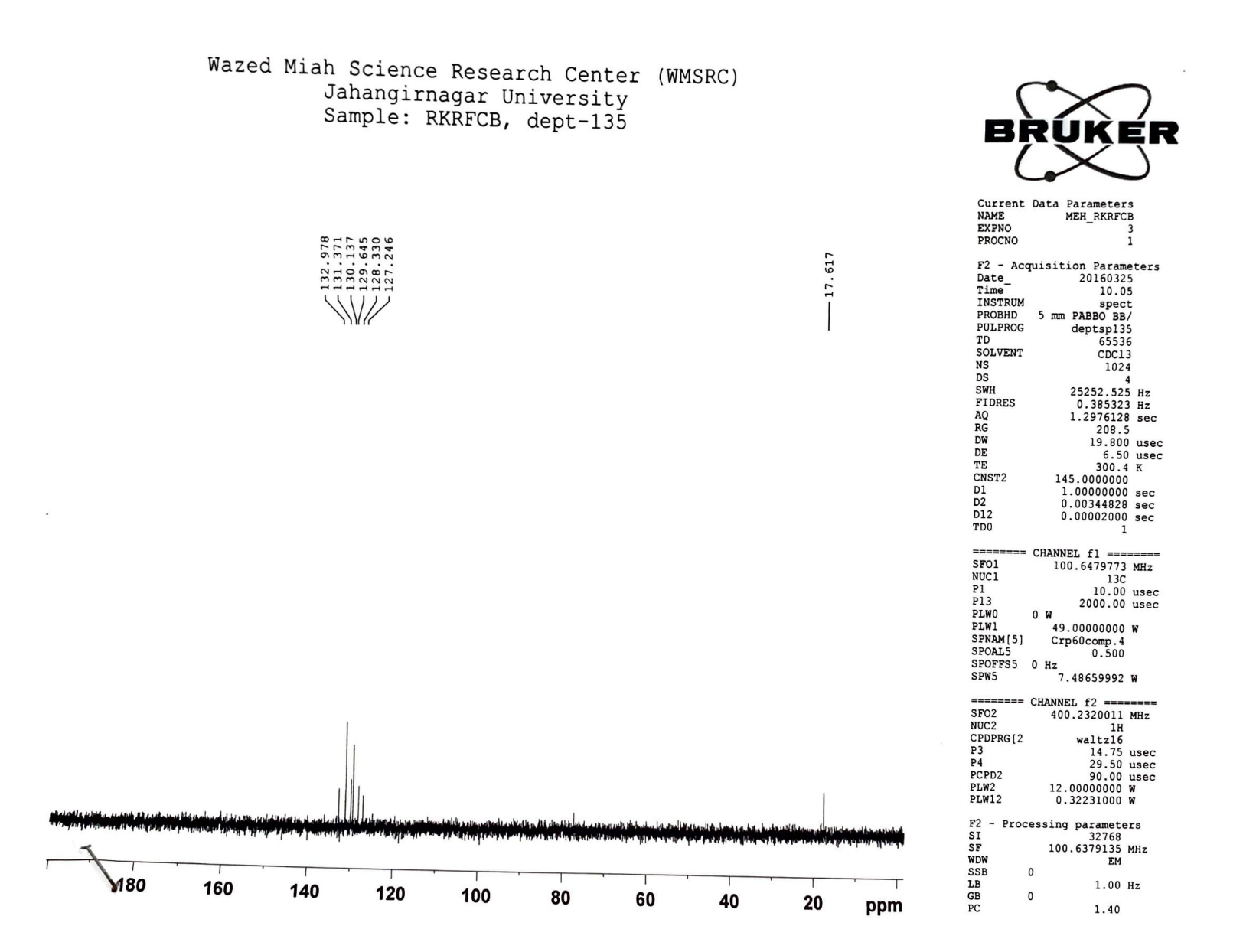

Supplement: S29 Fig — (DOCX) [file pone.0247619.s029.docx]

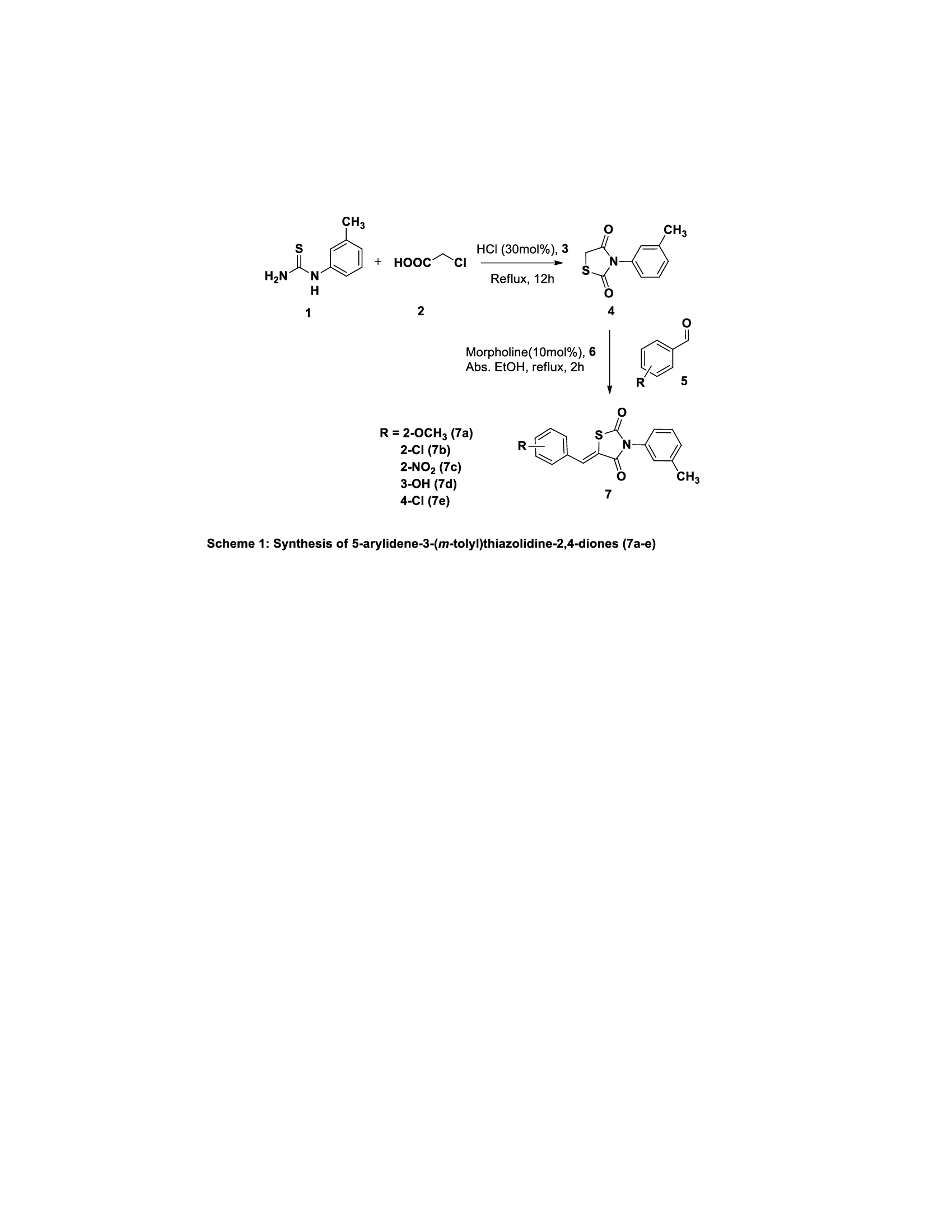

Supplement: S33 Fig — (TIF) [file pone.0247619.s033.tif]
