# Supplementary material for: A convenient approach to synthesize substituted 5-Arylidene-3-m-tolyl thiazolidine-2, 4-diones by using morpholine as a catalyst and its theoretical study
Source: PLoS One. 2021 Mar 4;16(3):e0247619. doi: 10.1371/journal.pone.0247619 (PMC7932548; doi:10.1371/journal.pone.0247619)
Supplement: S3 Fig — (DOCX) [file pone.0247619.s003.docx]

**S3 Fig: ^1^H-NMR Spectrum of 3-*m*-tolyl tiazolidine-2, 4- dione (4)**
