# Supplementary material for: A convenient approach to synthesize substituted 5-Arylidene-3-m-tolyl thiazolidine-2, 4-diones by using morpholine as a catalyst and its theoretical study
Source: PLoS One. 2021 Mar 4;16(3):e0247619. doi: 10.1371/journal.pone.0247619 (PMC7932548; doi:10.1371/journal.pone.0247619)
Supplement: S31 Fig — (DOCX) [file pone.0247619.s031.docx]

**S31 Fig:** Chemical structures of molecules optimized with DFT-B3LYP (6-31G, d) level of theory

| 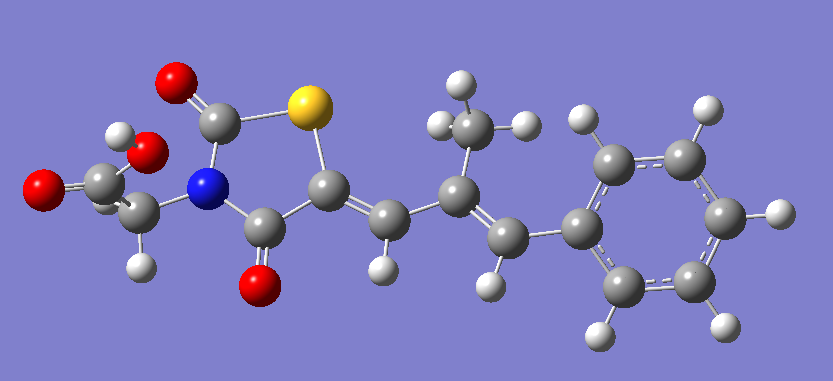  Epalrestat | 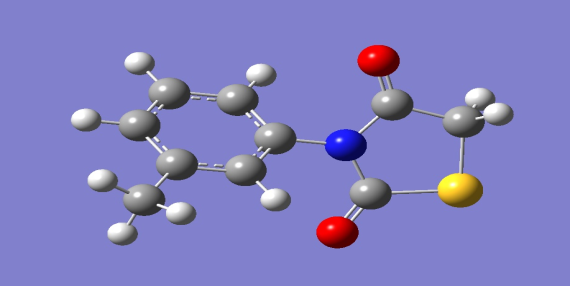  C_10_H_9_NO_2_S (4) |
| --- | --- |
| 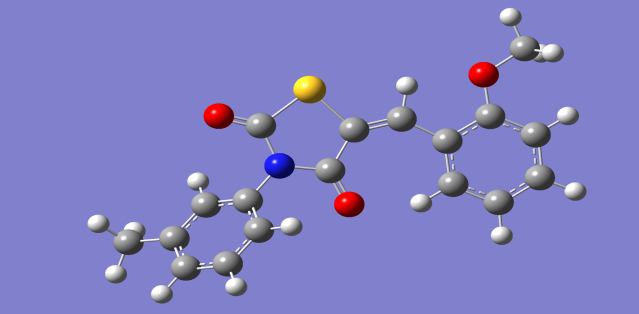  C_18_H_15_NO_3_S (7a) | 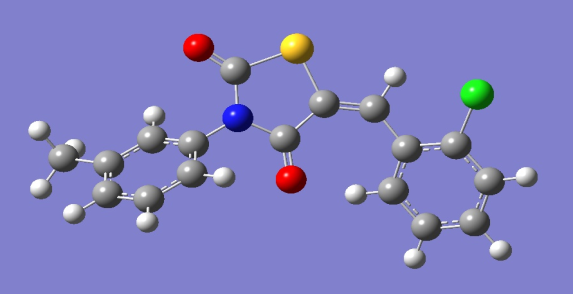  C_17_H_12_NO_2_SCl (7b) |
| 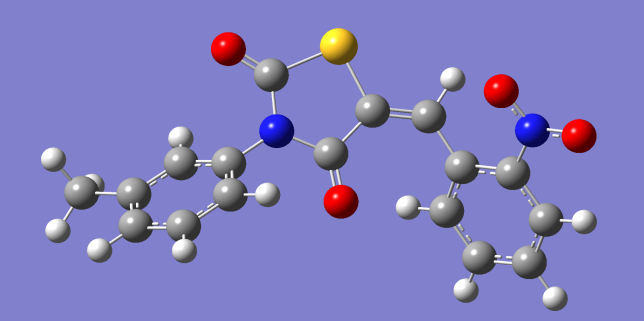  C_17_H_12_N_2_O_4_S (7c) | 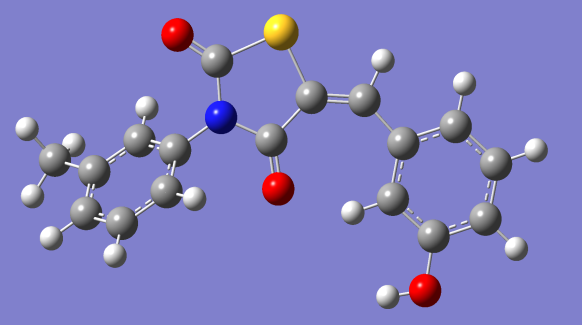  C_17_H_13_NO_3_S (7d) |
| 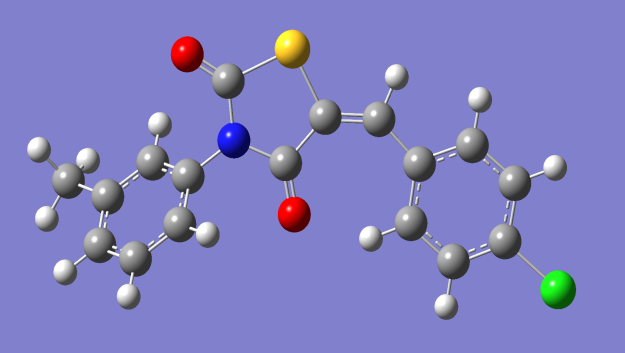  C_17_H_12_NO_2_SCl (7e) |  |
