# Supplementary material for: A convenient approach to synthesize substituted 5-Arylidene-3-m-tolyl thiazolidine-2, 4-diones by using morpholine as a catalyst and its theoretical study
Source: PLoS One. 2021 Mar 4;16(3):e0247619. doi: 10.1371/journal.pone.0247619 (PMC7932548; doi:10.1371/journal.pone.0247619)
Supplement: S32 Fig — (DOCX) [file pone.0247619.s032.docx]

**S32 Fig: HOMO and LUMO structures of the optimized molecules.**

| Compounds | HOMO | LUMO |
| --- | --- | --- |
| Epalrestat | 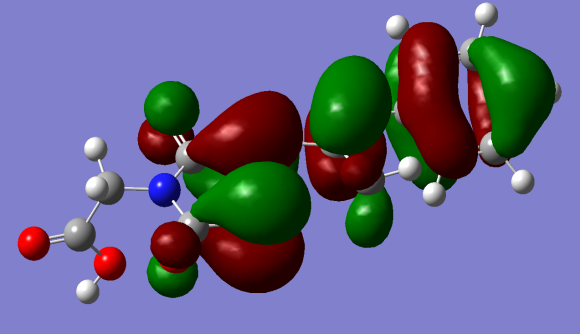 | 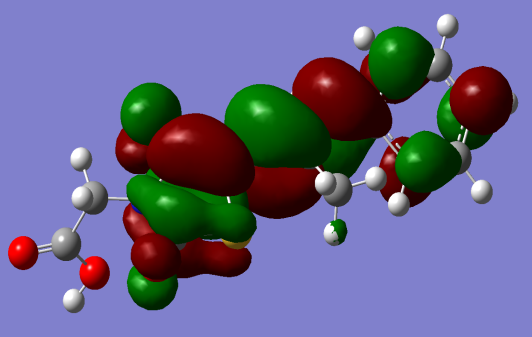 |
| 4 | 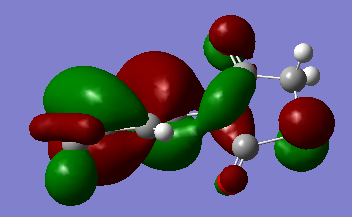 | 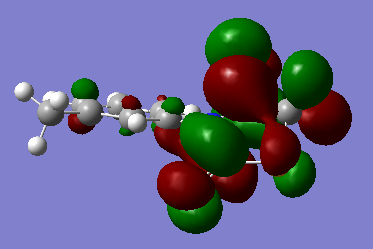 |
| 7a | 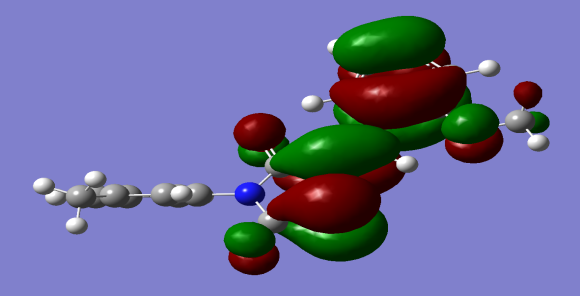 | 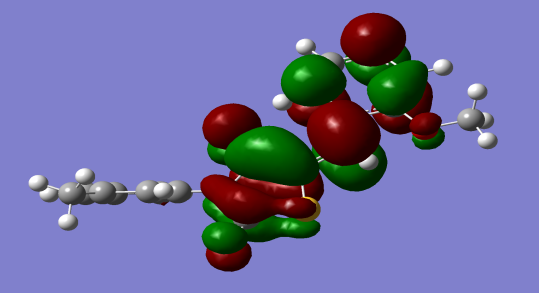 |
| 7b | 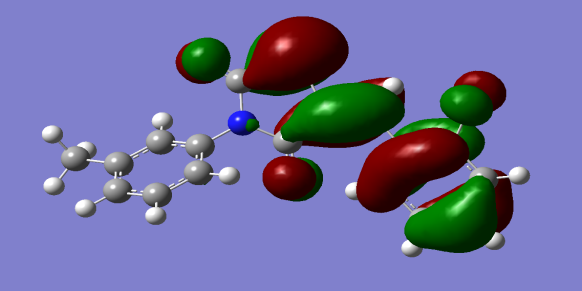 | 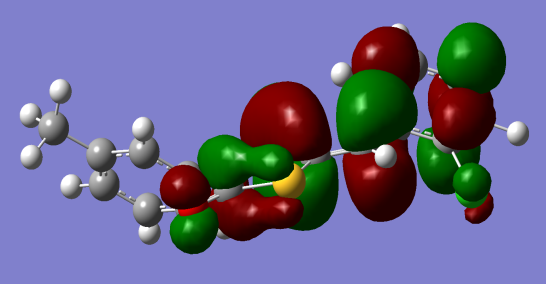 |
| 7c | 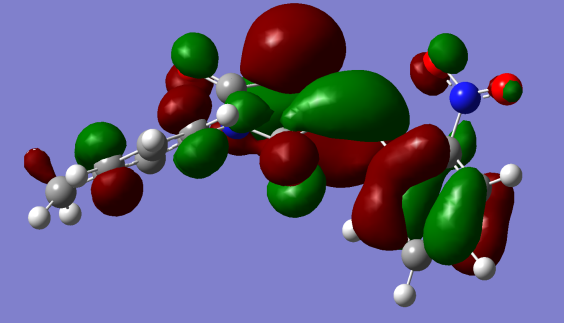 | 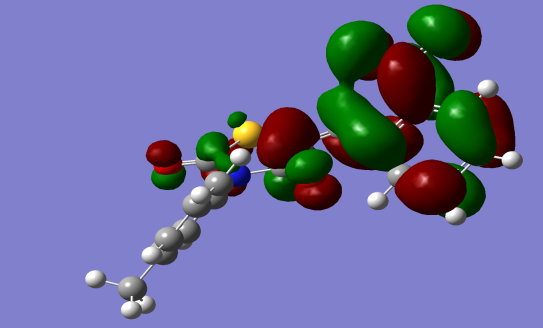 |
| 7d | 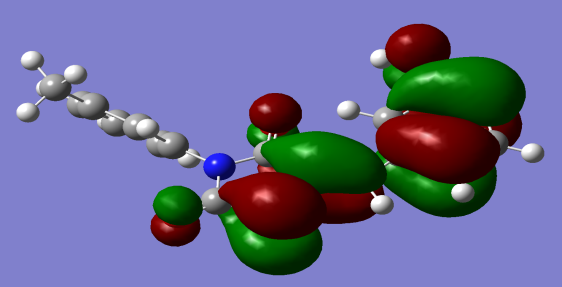 | 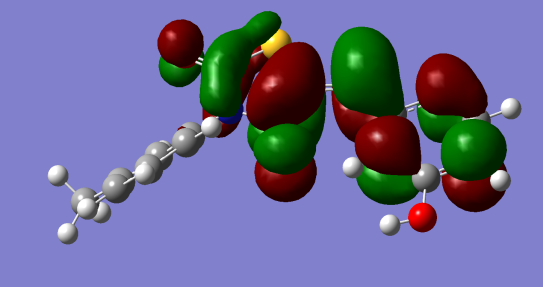 |
| 7e | 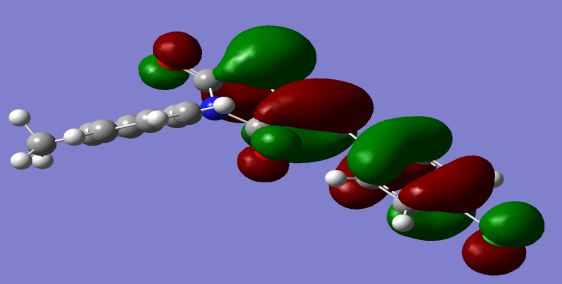 | 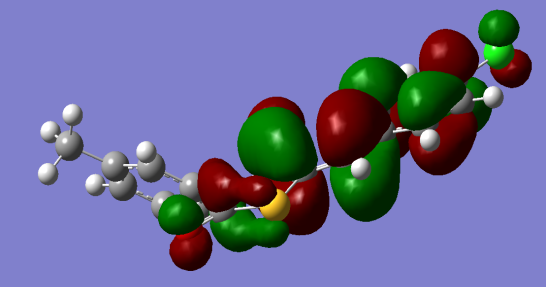 |
